# Inside the “African Cattle Complex”: Animal Burials in the Holocene Central Sahara

**DOI:** 10.1371/journal.pone.0056879

**Published:** 2013-02-20

**Authors:** Savino di Lernia, Mary Anne Tafuri, Marina Gallinaro, Francesca Alhaique, Marie Balasse, Lucia Cavorsi, Paul D. Fullagar, Anna Maria Mercuri, Andrea Monaco, Alessandro Perego, Andrea Zerboni

**Affiliations:** 1 Dipartimento di Scienze dell'Antichità, Sapienza Università di Roma, Rome, Italy; 2 School of Geography, Archaeology and Environmental Studies, University of the Witwatersrand, Johannesburg, South Africa; 3 McDonald Institute for Archaeological Research, University of Cambridge, Downing Street, Cambridge, United Kingdom; 4 Dipartimento di Biologia Ambientale, Sapienza Università di Roma, Rome, Italy; 5 Department of Anthropology, Washington University in St. Louis, St. Louis, Missouri, United States of America; 6 UMR 7209 “Archéozoologie, archéobotanique: sociétés, pratiques et environnements”, CNRS/MNHN, Paris, France; 7 Department of Geological Sciences, University of North Carolina, Chapel Hill, North Carolina, United States of America; 8 Laboratorio di Palinologia e Paleobotanica, Dipartimento di Scienze della Vita, Università di Modena e Reggio Emilia, Modena, Italy; 9 Dipartimento di Scienze della Terra “A. Desio”, Università degli Studi di Milano, Milan, Italy; University of Oxford, United Kingdom

## Abstract

Cattle pastoralism is an important trait of African cultures. Ethnographic studies describe the central role played by domestic cattle within many societies, highlighting its social and ideological value well beyond its mere function as ‘walking larder’. Historical depth of this African legacy has been repeatedly assessed in an archaeological perspective, mostly emphasizing a continental vision. Nevertheless, in-depth site-specific studies, with a few exceptions, are lacking. Despite the long tradition of a multi-disciplinary approach to the analysis of pastoral systems in Africa, rarely do early and middle Holocene archaeological contexts feature in the same area the combination of settlement, ceremonial and rock art features so as to be multi-dimensionally explored: the Messak plateau in the Libyan central Sahara represents an outstanding exception. Known for its rich Pleistocene occupation and abundant Holocene rock art, the region, through our research, has also shown to preserve the material evidence of a complex ritual dated to the Middle Pastoral (6080–5120 BP or 5200–3800 BC). This was centred on the frequent deposition in stone monuments of disarticulated animal remains, mostly cattle. Animal burials are known also from other African contexts, but regional extent of the phenomenon, state of preservation of monuments, and associated rock art make the Messak case unique. GIS analysis, excavation data, radiocarbon dating, zooarchaeological and isotopic (Sr, C, O) analyses of animal remains, and botanical information are used to explore this highly formalized ritual and the lifeways of a pastoral community in the Holocene Sahara.

## Introduction

### Stone monuments, rock art and cattle: an African legacy

Early Holocene cattle-based pastoralism is the oldest form of productive economy in Africa, which precedes agriculture [1], [2]. Despite the idea of an independent African domestication of *Bos primigenius* remains still controversial [3], [4], a genetic input of African aurochs during the long and discontinuous domestication process is possible [5]. Timing and mechanisms of livestock spread in Africa have been studied primarily combining radiocarbon dates of morphologically domestic remains with specific regional trajectories [6]. Secondary exploitation of cattle appears much later, with the earliest evidence of dairying from the central Sahara at around 6100 BP [7].

Notwithstanding ecological barriers and diseases such as trypanosomes [8], cattle pastoralism spread all over the continent, becoming a momentous segment of African economy and society. Even today, relations between herders and their animals, especially in Eastern Africa, are particularly strong and well beyond the mere use of cattle as ‘walking larder’ [9]. Travellers, explorers and ethnographers of the 19^th^ and early 20^th^ century gave vivid narratives about the crucial importance of cows and bulls: Herskovits [10] coined the concept of “African Cattle Complex”, underlining the role of these animals within many African populations.

Bovines represent the primary wealth and are often used to pay bride and blood fines, being the basis for social prestige. Only rarely eaten, their slaughtering is often strongly socialized and special places are required for this purpose e.g., [11]–[13].

There is therefore scarce doubt that cattle exploitation and pastoral identity in Africa largely overlap e.g., [4], [11]–[16] and roots of this African legacy must be found in its remote past.

Given the extraordinary historical depth of cattle management in Africa, it is not a surprise that most archaeological investigations focussed on defining nature and organization of African pastoralists [6], [17]. However, the exploration of ideological and ritual aspects was mostly directed towards the study of human mortuary practices [18], [19], monumental architecture [20], [21] and rock art [22], [23]. Yet, cattle and pastoral activity are obsessively present in African iconography: in the Sahara, more than 60% of art panels portrait cattle or cattle-related scenes [24].

Artworks of bovidian/pastoral style are thus the tangible evidence of a shared heritage ideologically focussed on cattle. However, problems in its dating [25] make it difficult to relate settlement and subsistence data with the Saharan pastoral ideological world.

Another important archaeological evidence of cattle centrality in the African prehistoric pastoral world is represented by stone monuments with articulated or disarticulated remains of bovines, repeatedly interpreted as the expression of the “African Cattle Complex”, such as those of Nabta Playa in Egypt [26] or Adrar Bous in Niger [27]–[29]. More recent research relates the presence of accumulations of cattle bones, defined by the authors “Tenerian meals”, to feasting activities [30]. Further contexts with possible ritual depositions of cattle are reported from Talak–Timenrsoi in western Air, Niger, and dated between 5400 and 4800 BP [28]. The site of Mankhor, in the Algerian Tadrart, dated between 5525 and 4865 BP, shows evidence of ritual deposition [31]. The ritual interment of cattle remains appears however to be a long standing *habitus*, as testified by other Niger sites dated as late as 3500 BP [28], plus for example the evidence from the Nile valley e.g., [32].

**Figure 1:**
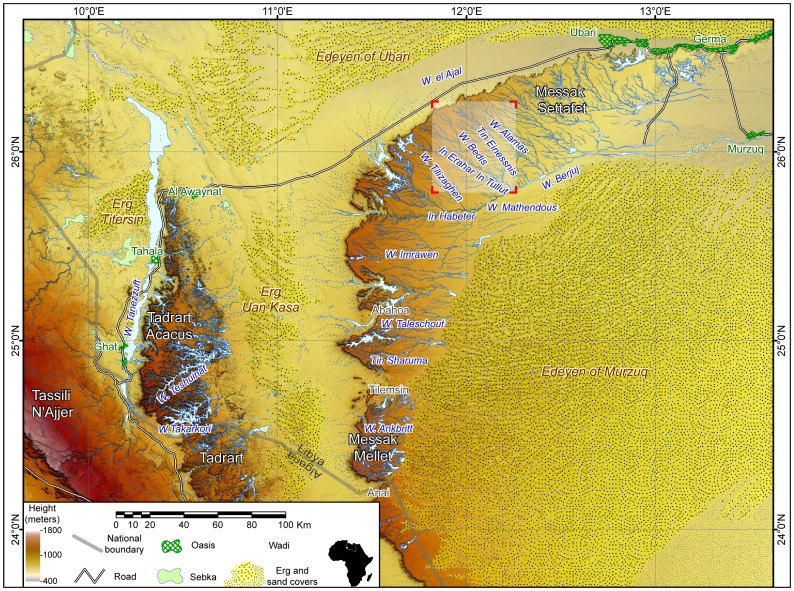
The Messak plateau and surroundings. The white insert shows the area of fieldwork (2000; 2007–2010).

**Figure 2:**
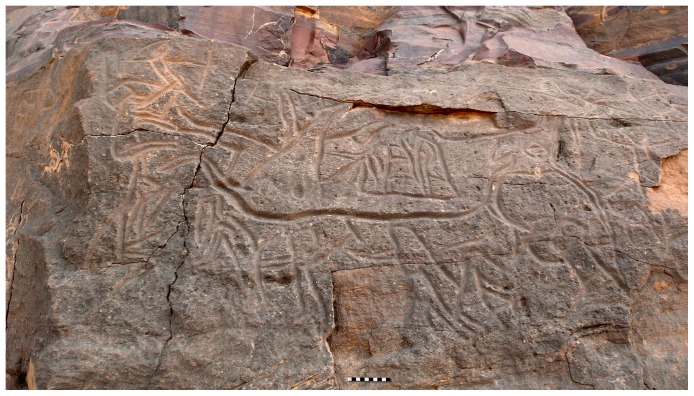
The sacrifice of a bull at In Erahar. The *corbeille* 07/110 C1 is just above the engraved wall: it yielded the remains of a bull, with offerings of flowers, a pot and the stone maces possibly used to kill the animal.

Cattle, stone monuments and rock art appear to be important elements of African prehistoric pastoral societies, but they rarely occur together so as to be multi-dimensionally explored: the Messak plateau (SW Libya) in the central Sahara represents an outstanding exception (Fig. 1). Here, engravings portraying pastoral activities–which include the vivid representation of cattle sacrifices (Fig. 2)–are common and often in spatial relations with stone monuments. Some of these structures were already excavated in the 1990s [33]: they revealed the existence of deliberate depositions, mainly of cattle, with engravings of bovines strictly associated. A specific project was later launched (Messak Ceremonial Monuments Project, MCMP, 2007–2010) with the aim to explore the complexity of this cultural phenomenon, either in time or space.

This has been achieved through a campaign of surveys and excavations of stone monuments and associated rock art, combining environmental information, GIS spatial analysis, archaeological data, radiocarbon chronology, zooarchaeology, archaeobotany and isotopic investigation.

### Middle Pastoral herders of the central Sahara

Cattle and small livestock were introduced in the Central Sahara at the end of the 8^th^ millennium BP, and slowly adopted by local groups of hunter-gatherers [2]. In the Acacus and Messak mountains (SW Libya) a full exploitation of domesticates, which included dairying [7], is dated to the Middle Pastoral (6100–5000 BP), a cultural phase generally characterized by wet and warm environmental conditions [34]–[36].

Past geoarchaeological surveys and excavations helped to define settlement system, food security, mortuary and social practices of Middle Pastoral groups [37]–[39]. This was mainly achieved in the Acacus massif and neighbouring dune fields (Erg Uan Kasa), thanks to the good state of preservation of stratigraphic contexts in the mountain range, which yielded a rich archaeological record [37]. The territorial scale of analysis also allowed for the understanding of mobility patterns between different ecological niches of these cattle herders [38]–[40], which were regulated by the high seasonality of monsoonal precipitation recorded for the middle Holocene [34]. Large and semi-residential sites are abundant along the former shores of lakes in the dune fields; they were likely occupied during the rainy season (summer), while during the drier season (winter) herders concentrated in the mountain range, as also indicated by pollen data [35].

The Acacus-Uan Kasa model might also apply to the Messak plateau and the vast Edeyen of Murzuq, where solid locational relations might have linked the two areas. In the latter, several rich Middle Pastoral sites were mapped and some were excavated [37], [41]. They can be reasonably interpreted as summer semi-residential sites, exploiting the water-rich areas surrounding the lakes [34]. Unlike for the Acacus-Uan Kasa system, we found poor evidence of Holocene settlement in the Messak, with a dozen of Middle Pastoral contexts, generally showing ephemeral and light occupation [42], [43]. Along with dated sites, a large number of contexts lack chronologically diagnostic features: some or many of them could be of Middle Pastoral age. As a general tendency however, no Holocene settlement site, regardless of its chronological attribution, shows complex and articulated features. In this sense, the paucity of settlements (be it numerical or in terms of complexity) contrasts with the richness of stone ceremonial monuments [33], [44]–[49], rock art [47]–[49] and quartzarenite quarrying [50]. Many of these archaeological contexts might belong to the Middle Pastoral, as recently proposed for the “Messak school” engravings [25].

### The Messak. Environmental and archaeological background

Our study area is a large plateau which extends over more than 15,000 km^2^ between 24° and 26° 30′ latitude N, and 11° and 13° longitude E [51]. It can be divided into two adjoining regions separated by the Tilemsin corridor: Settafet (‘black’, in local language) and Mellet (‘white’). The Messak is a cuesta type massif cut into the Jurassic to Cretaceous Messak Sandstone, gently tilted eastward and delimited by an abrupt scarp. The maximum altitude is 1200 m asl. A dense network of fossil wadis with a dendritic pattern dissects the plateau, originated in the Tertiary under a pluvial climate. The present climate of the region is hyperarid: mean annual temperature is 22°–25 °C; mean annual rainfall is 0–10 mm. Both climate and palaeoclimate depend on low altitude pressure and winds over the continent and the seasonal migration of the Intertropical Convergence Zone, resulting in belts of monsoonal climate with summer rains and dry winters [52].

The flora is still not fully known, but most of the species described for the central Sahara [53] were observed in the field. Acacias include *Faidherbia albida*, *Acacia tortilis* and *A.nilotica*. Shrubs of *Cornulaca monacantha, Pulicaria crispa*, *Panicum turgidum* and *Spipagrostis pungens* are common. Desert savannah and Saharo-montane vegetation, typical of the Saharan Transitional zone [54], is prevalent in the wadis.

The main physiographic units of the massif correspond to residual surfaces (hamada and serir), solutional depressions, slope deposits, and a composite escarpment [51]. The typical landscape of the Messak plateau is the black hamada surface, whose clasts are coated by a dark Mn-rich varnish [55], interrupted by wide serir spots. The desert pavement overlies relict and complex rubified paleosols, which are discontinuously present on the plateau. These formed under pluvial phases since the early Pleistocene. The most recent pedogenesis is dated to the middle Holocene [56].

The hamada is now a palimpsest of lithic scatters dating from Early Stone Age to historical times [43], [57]. Holocene occupation features hundreds of funerary and ritual structures [33], [43], [44], whereas, as already emphasized, only light and ephemeral dwellings were recorded [37], [42], [43], [58], [59]. Rare deposits are preserved in rock shelters [60]. The most impressive Messak feature is rock art: the wadi areas are dotted by thousands of engraved panels of Holocene age [25], [47]–[49], [61], [62].

### The Messak Ceremonial Monuments Project (MCMP)

The first monuments with animal remains were found during a rescue operation to assess damages caused during oil prospecting [33], [42]. These added to the results of the excavation of a standing stone located at In Habeter, also containing cattle remains [63]. Such features suggested the existence of ceremonies clearly connected to pastoral rituals focussed on cattle ideology [33].

To assess the extent of the phenomenon, and to define its nature and meaning, we launched the “Messak Ceremonial Monuments Project” (MCMP 2007–2010).

The research has been carried out within the activities of The Archaeological Mission in the Sahara, Sapienza University of Rome and the Department of Archaeology (DoA), Tripoli, directed by SDL. All necessary permits were obtained for the field studies and laboratory analyses (including destructive ones) presented here.

## Results and Discussion

Our data convey to suggest that during the middle Holocene (6080–5120 BP or 5200–3800 BC) the Messak plateau homed the highly formalized local expression of a wider ideological phenomenon centred on domestic cattle. The slaughtering of bovines was an impressive enduring ritual, which should be considered as a central part of the socio-cultural system of Messak Middle Pastoral herders.

The arguments to support our interpretation combine different territorial scales of analysis (from regions to monuments) and involve several perspectives: GIS analysis of *corbeilles* and rock art contexts (including their geomorphological setting); archaeological excavations; radiocarbon dates on animal bones and/or associated contexts; classification of archaeological materials (pottery, lithics); zooarchaeological analysis; isotopic data (^87^Sr/^86^Sr, δ^13^C, δ^18^O) on faunal remains; botanical information, as described below.

### Corbeilles and rock art: a GIS approach

The sources for our GIS platform are published and unpublished information, together with our fieldwork data, for a total of 197 structures (Text S1; Table S1). Depending on the different sources, quantity and quality of data for each structure can vary from a simple positioning to a full excavation record. To overcome this heterogeneity, the analyses were first performed on the spatial location of the structures, also using satellite imagery: for each structure we have analysed topographical and geomorphological setting, together with the hierarchy, geography and morphology of the related wadi (Table S1).

**Figure 3:**
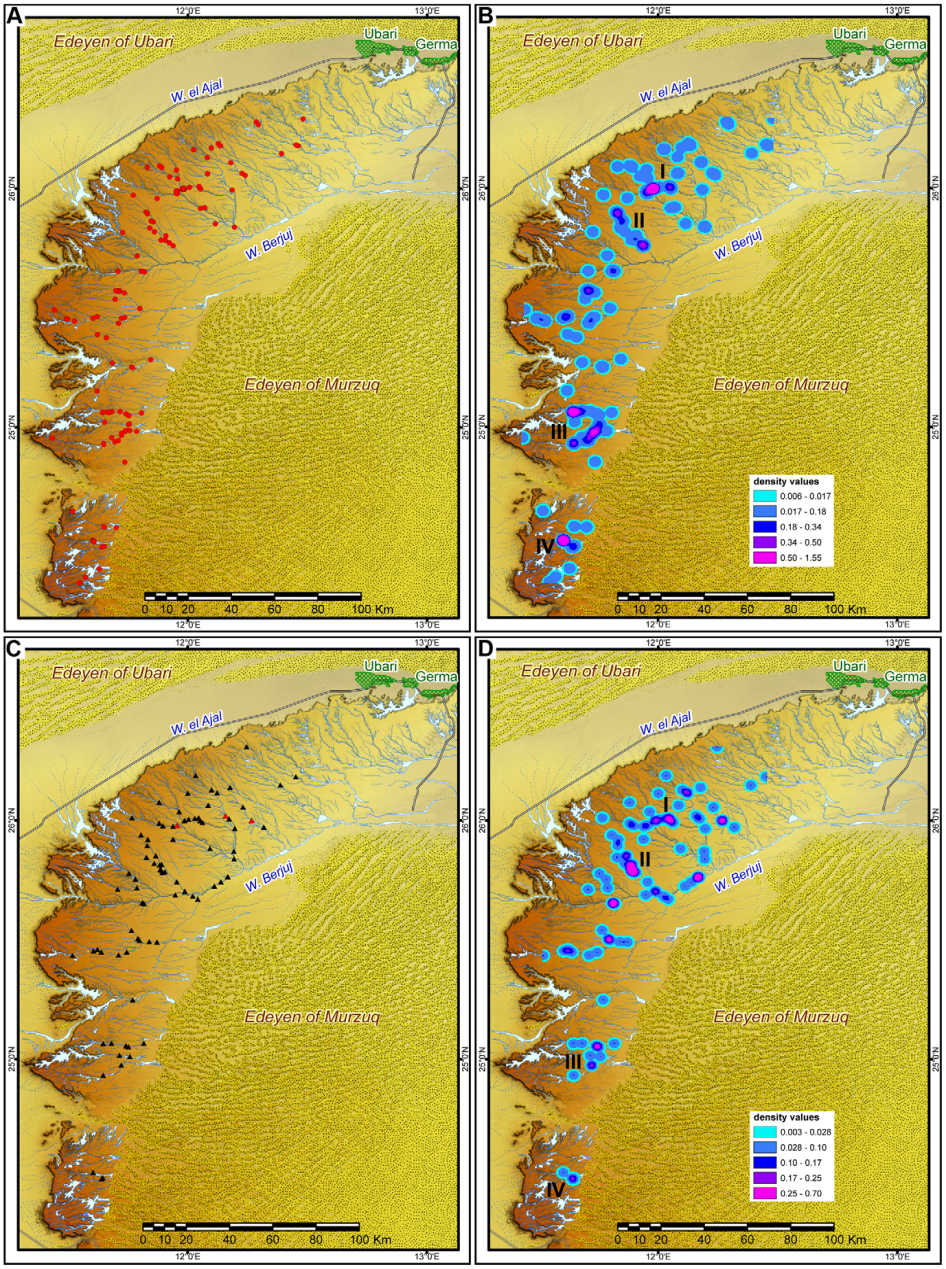
Desktop and GIS analysis. Distribution maps of *corbeilles* (A) and Middle Pastoral engravings (C–red triangle indicates the artworks depicting the slaughtering of cattle) and their density analysis (B, D). The four densest clusters are indicated (I–IV).

The distribution map proves the widespread presence of *corbeilles* all over the region, but for the north-eastern Settafet (Fig. 3). The structures are mainly located in specific and recurrent locales: they are placed on the hamada (79%), along the middle courses of the principal wadis (79%), in correspondence with their widest meanders (86%) and close to the wadi bank (<75 m, 83%).

The geomorphology of these locales corresponds to valleys with flat floors and steep sides [51]. Wadi cuts are mostly attributed to groundwater seepage erosion; it is likely that these parts of the wadis experienced high water availability thanks to groundwater coming to light a few hundreds of meters ahead. These places thus represented favourable areas for grazing and water supply in an otherwise harsh landscape.

The minimum reciprocal distance between monuments, the distance of the structure(s) from the wadi banks and the accessibility to the ancient river are important elements to define the micro-topographic features of these contexts. *Corbeilles* are commonly very close to one another (ca. 41% under 30 m), creating ‘aggregate’ contexts. Interestingly, very remote and isolated structures are not rare (> 15%).

The striking proximity between monuments is mirrored by the analysis of the Average Nearest Neighbour, which shows a high index of clustering (Observed Mean Distance 1546.52 m; Expected Mean Distance 4125.53 m; NN ratio 0.37; z Score −16.79; p-value 0.0000), highlighting the non-random distribution of the structures all over the region and validating the regional organization of the cultural phenomenon. Analysis of density of *corbeilles*' location, based on the kernel method [64], identifies the existence of four areas with very high clustering, located respectively at Wadi Bedis and Wadi Tilizaghen (Northern Settafet); Wadi Taleschout -possibly being part of a wider cluster together with Tin Sharuma- (Southern Settafet); and Wadi Ankbritt (Mellet). Although the chronological relationships between the monuments cannot be ascertained on the basis of survey information alone, the presence of many and very similar structures in specific locales should be in any case interpreted as evidence of important places in the pastoral landscape.

Most interestingly, none of the architectural features of the *corbeilles* (size, elevation, building elements, presence and type of standing stone) show significant distribution in the landscape: this reinforces the value of the *corbeille* itself (and not of its building elements) as a codified landmark in the Messak Middle Pastoral world.

The distribution of the *corbeilles* and particularly that of the main concentrations of monuments largely matches that of rock art. Combining different sources on rock art contexts, we identified 102 scenes (Table S3) clearly referable to a Middle Pastoral phase (following [25]), whose spatial distribution and density are extremely similar to those of the *corbeilles* (Fig. 3). In these rock art scenes, cattle is obsessively present as an isolated subject or as part of complex scenes referring both to everyday life duties and more symbolic settings. Interestingly, the only three artworks depicting the slaughtering of cattle are all located in the northern area, where one of the most significant concentrations of *corbeilles* occurs (at least in one case, 07/110 C1, the structure with cattle remains is located immediately above the engraving).

### Survey in the Northern Messak Settafet

**Figure 4:**
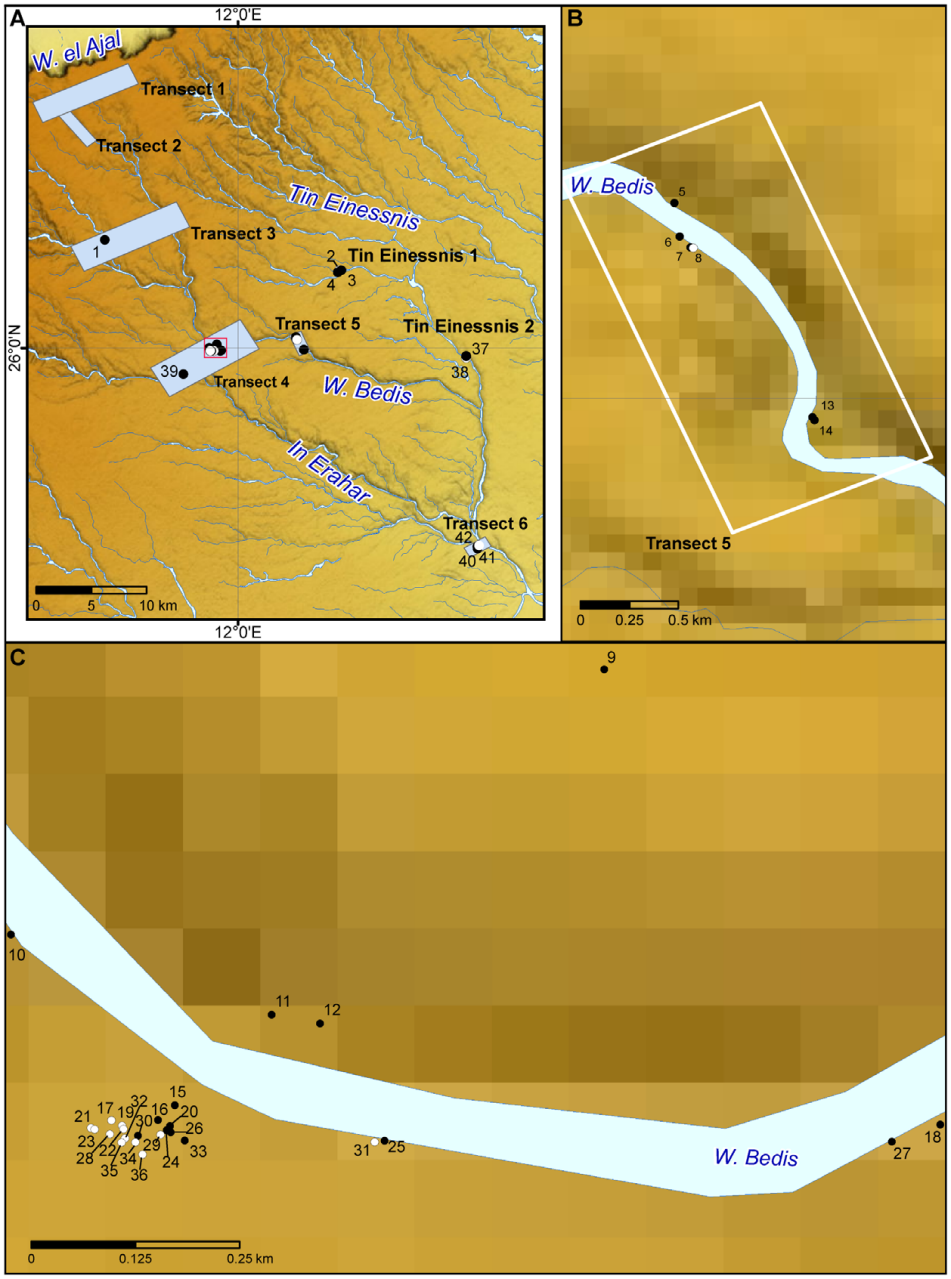
Detail of the area of intensive survey. General area (A); detail of Transect 5 (B); magnified view of red square in Transect 4 (C). The excavated monuments are indicated by full dots (black for the *corbeilles*, white for the other monuments) and their Id number (see Table 1 for details).

The rationale of the MCMP fieldwork was to assess the distribution of stone monuments and to investigate their correlations with the landscape. A series of sampling areas were set along an ideal N–S transect intercepting the main geographic and physiographic units, following the course of one of the main fossil hydrographical arteries of the Messak (In Tullult, In Erahar, Wadi Bedis: Fig. 4; Text S2). This fieldwork adds to research undertaken in the 1990s in the areas of Tin Einessnis (1 and 2) and In Habeter III (see [33]).

A total surface of approximately 75 km^2^ was investigated, with 219 Holocene archaeological contexts identified (Text S2; Table S2). Most of them are conical tumuli, followed by stone structures and other stone features: the *corbeilles* are 34. The chronological or cultural attribution is difficult: most of the contexts are generically referred to Pastoral age; many structures are of recent, historical occupation. The contexts attributed to the Middle Pastoral are a few dozens.

**Figure 5:**
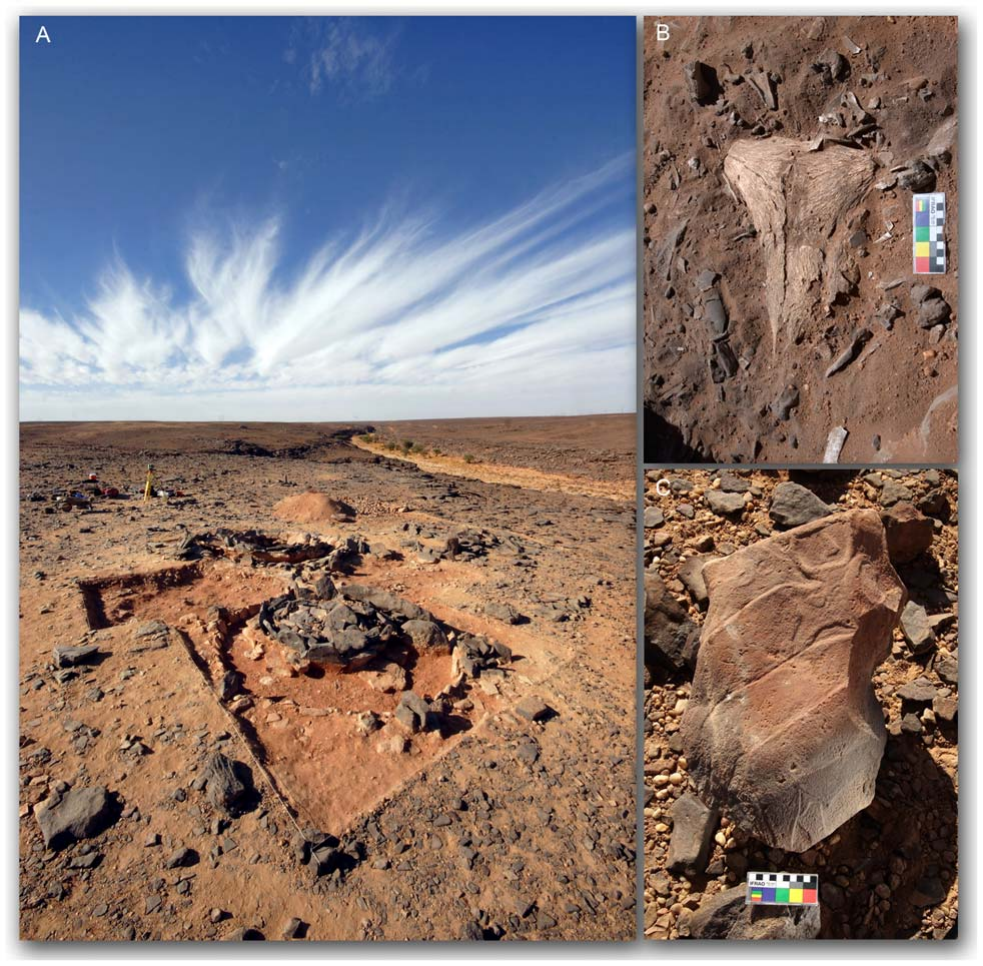
Examples of excavated archaeological features. View of the excavations at 07/39 C2 and C3 (A), with detail of the skull from C3 (B). From monument 07/39 C1, the engraved boulder reused as trapping stone and then as building material (C).

However, it is clear that some locations, i.e. the most (and probably the few) geomorphologically favourable places for grazing and water supply, assumed a key role for the cultural and ritual activities of the Middle Pastoral herders, and were reoccupied and reused by later pastoralists. This evidence is also supported by the high concentration in a few areas of trapping/tethering stones (TS): these stones, generally represented in rock engravings as hunting devices e.g., [48], are made of slabs or boulders of different size (up to 1 m) with notches or grooves to block a rope. Even if reused over time (Fig. 5), the very large quantity of these stones in places clearly unsuitable for hunting activity -such as the area of Tin Einessnis I (256 TS) and the Bedis meander (around 07/39 and 07/40: 187 TS; around 07/68: 126 TS)-, suggests a functional interpretation of these stones as tethering elements for domestic animals. Should they either represent the archaeological evidence of the gathering of several people (as potentially suggested by the presence of ceremonial monuments) or the reuse over time of the same place, the outcome is the same: these concentrations of tethering stones mark, together with the densest clusters of ceremonial monuments, locales of social importance and enduring value for Messak pastoral groups.

### The excavations of stone monuments

As a whole we excavated 42 stone monuments, mostly in Wadi Bedis meander. Most of the monuments were *corbeilles* (26), together with stone structures (7), tumuli (4), stone platforms (2) and other elements (Table 1; Fig. 5): when monuments were very close to each other, extended excavations or test pits were carried out to assess their function and possibly verify the chronological correlation.

**Table 1:** Main features of excavated contexts with evidence of rituals related to animals, sorted by geographic position.

Id	Structure	Type	Annex	Standing stone	Age C14 BP	Fauna	Archaeological Materials		Rock art associated with the structure*
							*Stone tools*	*Pottery*	
1	07/83	simple *corbeille*	—	1 central	—	—	—	—	—
2	00/301	deflated tumulus	2	—	6080±80	*Bos taurus*, *Ovis* vel *Capra*	3 maces, 1 gouge	1 potsherd, rocker plain edge (Middle Pastoral)	1) two cows and one enigmatic subject; 2) a foot
3	00/301a	simple *corbeille*	—	—		—	—	—	—
4	00/300	complex *corbeille*	—	1 central	5610±40	Large ungulate	1 bifacial tool	—	—
5	08/25	simple *corbeille*	—	—		—	—	—	—
6	09/69 C2	simple *corbeille*	—	—		—	—	—	—
7	09/69 C1	simple *corbeille*	—	—	5350±25	*Bos taurus*	1 mace in the structure and another one between C1 and C2; 1 bifacial tool	—	—
8	09/69 T1	deflated tumulus	—	1 on the NE side		—	—	—	—
9	07/28 C1	simple *corbeille*	—	1 central	5330±30	*Bos taurus*	1 mace close to the structure	1 undecorated potsherd	—
10	07/79 C1	double ring *corbeille*	—	1 central	5400±30	*Bos taurus*	2 bifacial tools	1 undecorated potsherd	—
11	07/68 C1	simple *corbeille*	—	1 central	5350±25	undeterminable	1 pick	—	—
12	07/59 C1	complex *corbeille*	—	1 external		—	—	—	1) undeterminable; 2) schematic bovid
13	08/01 C2	simple *corbeille*	—	1 central		—	—	—	—
14	08/01 C1	simple *corbeille*	—	1 central	5220±30	undeterminable	—	—	—
15	07/39 C6	simple *corbeille*	—	1 collapsed	5660±30	undeterminable	—	—	—
16	07/39 C5 *corbeille*	complex *corbeille*	—	1 central; 1 external (see below)	5200±30	*Bos taurus*	—		—
16a	07/39 C5 ext. stele	standing stone	—	external		*Ovis* vel *Capra*	—		—
17	07/40 SS22	stone structure	—	—	1790±25	undeterminable	—	—	—
18	07/10 C1	complex *corbeille*	6	9: 1 central; 1 for each annexes; 2 external	5490±30	undeterminable	—	—	—
19	07/40 SS18	stone structure	—	—		—	—	—	—
20	07/39 C4 *corbeille*	complex *corbeille*	1	1 central; 1 in the annex (see below)	5340±40	*Ovis* vel *Capra*	—	2 potsherds, rocker plain edge (Middle Pastoral)	1) main figure of a bull, surrounded by at least two other smaller bovines, of Pastoral style; 2) an enigmatic engraving probably representing two horns
20a	07/39 C4 ext. stele	standing stone		external		*Ovis* vel *Capra*	—	—	muzzles of two antelopes in profile of Pastoral style
21	07/40 SS40	stone structure	—	—		—	—	—	—
22	07/40 SR17	stone ring	—	——	2980±25	—	—	—	—
23	07/40 SP11	stone platform	—	—		—	—	—	—
24	07/39 C3	double ring *corbeille*	—	1 central	5520±30	*Bos taurus*, small ungulate	2 maces	2 potsherds, Alternatevily Pivoting Stamp (Middle Pastoral)	1) a barely recognisable subject–probably an antelope
25	07/37 C1	simple *corbeille*	—	1 central		undeterminable	1 mace close to the external perimeter	—	—
26	07/39 C2 *corbeille*	complex *corbeille*	—	1 central collapsed; 5 external		undeterminable	—	6 potsherds, rocker plain edge (Middle Pastoral; 3 potsherds, Alternatevily Pivoting Stamp return tecnique (Middle Pastoral); 1 undecorated	—
26a	07/39 C2 ext. area	area adjacent to the perimetral external wall	—	—		*Bos taurus, Ovis* vel *Capra*		—	—
27	07/11 C1	simple *corbeille*	—	1 collapsed		—	—	—	—
28	07/40 SR13	stone ring	—	—		small ungulate	—	—	—
29	07/39 SS1	stone structure	—	—		—	—	—	—
30	07/40 C1	simple *corbeille*	—	1 central	5400±30	*Bos taurus*, *Ovis* vel *Capra*	1 mace	—	—
31	07/37 T1	deflated tumulus	—	—		—	—	—	—
32	07/40 SS16	stone structure	—	3 central		—	—	—	—
33	07/39 C1	simple *corbeille*	—	—	5190±30	*Equus* sp., small ungulate	—	—	1) an elliptical shape (fish?); 2) four cattle vertically superimposed of Pastoral style
34	07/40 SS15	stone structure	—	—		—	—	—	—
35	07/40 SR5	stone ring	—	—		—	—	—	—
36	07/40 SS 1a	stone structure	—	—		—	—	—	—
37	00/557	stone platform	—	—	5750±40	Large ungulate	—	37 potsherds, rocker plain edge (Middle Pastoral); 10 undecorated potsherds; 12 undet.	—
38	00/556 *corbeille*	complex *corbeille*	—	1 central; 1 external (see below)	5150±110	*Bos taurus,* small ungulate	1 mace	32 undecorated potsherds; and 3 rocker plain edge decorated potsherds (Middle Pastoral)	1) an ovoid representation; 2) two superimposed cows of Pastoral style
38a	00/556 ext. stele	standing stone	—	external	5290±40	*Bos taurus,* small ungulate	—	—	two superimposed cows, of Pastoral style
39	07/110 C1	simple *corbeille*	—	1 central	5380±25	*Bos taurus*	2 maces inside the structure, 1 mace close to the external perimeter; 1 arrow head; 1 gouge	34 potsherds (partially refitting: rocker plain edge (Middle Pastoral)	1) schematic bovid; 2) ostrich of Pastoral style
40	07/55 C2	complex *corbeille*	8	—	5590±25	—	—	—	—
41	07/55 C1	complex *corbeille*	6	1 central; 1 easternmost annex	5570±25	*Bos taurus*	1 mace; 1 upper grinding stone with ochre	—	—
42	07/55 T1	deflated tumulus	—	—		—	—	—	—

* Unless specified, all engravings are of long lasting unclassifiable style

In sum, 22 between structures and associated features yielded faunal remains; stone tools or potsherds are present in 15 contexts; and 9 monuments shown slabs/boulders with rock art engravings.

Twenty-two structures were radiocarbon dated (on charcoal, charred animal bones), indicating a time span for the animal burial phenomenon in the region between 6080 and 5120 BP (approximately 5200–3800 BC: Table 2). The most ancient date refers to structure 00/301, a small deflated tumulus with animal remains coming from distinct fire points, very close to an empty, but not datable, *corbeille* (00/301a). The earliest date for a *corbeille* itself comes from structure 07/39 C6, dated to 5660±30 BP. The dates cluster between approximately 5400 and 5200 BP. Two dates on small features, both from Wadi Bedis meander, are much later and point to Final Pastoral and Garamantian visits, reinforcing once more the key role of these specific places over the centuries.

**Table 2:** Radiocarbon measures from excavated contexts (calibration: Oxcal online 4.1).

Structure	Lab. Code	Material	Age uncal BP*	Cal BC/AD (95.4% conf.)	Cal BP (95.4% conf.)	d13C,%
00/301	GX-28456	charcoal	6080±80	5216–4796	7165–6745	−23.9
00/557	GX-28448 AMS	charred bone	5750±40	4703–4500	6652–6449	−15.7
07/39 C6	UGAMS 3760	charred bone	5660±30	4553–4374	6502–6323	−10.19
00/300	GX-28457 AMS	charred bone	5610±40	4521–4356	6470–6305	−17.3
07/55 C2	UGAMS 5860	charcoal	5590±25	4462–4356	6411–6305	−10.6
07/55 C1	UGAMS 5859	charcoal	5570±25	4453–4355	6402–6304	−25.3
07/39 C3	UGAMS-3758	charcoal	5520±30	4450–4331	6399–6280	−23.46
07/10 C1	UGAMS 3756	charred bone	5490±30	4445–4262	6394–6211	−11.51
07/39 C4	UGAMS-2839	charred bone	5430±40	4355–4176	6304–6125	−16.74
07/40 C1	UGAMS 3761	charcoal	5400±30	4339–4085	6288–6034	−25.86
07/79 C1	UGAMS 3762	charcoal	5400±30	4339–4085	6288–6034	−23.51
07/110 C1	UGAMS 5853	charcoal	5380±25	4331–4076	6280–6025	−24.1
07/68 C1	UGAMS 5855	charcoal	5350±25	4321–4054	6270–6003	−25.7
09/69 C1	UGAMS 5856	charcoal	5350±25	4321–4054	6270–6003	−27.8
07/28 C1	UGAMS 5858	charcoal	5330±25	4245–4050	6194–5999	−26.2
00/556 ext. st.	GX-28447 AMS	charred bone	5290±40	4239–3992	6188–5941	−12.6
08/01 C1	UGAMS 3763	enamel bioapatite	5220±30	4223–3964	6172–5913	−4.45
07/39 C5	UGAMS 3759	charred bone	5200±30	4048–3960	5997–5909	−13.92
07/39 C1	UGAMS-3757	charred bone	5190±30	4044–3959	5993–5908	−13.79
00/556 corb.	GX-28446	charred bone	5150±110	4237–3707	6186–5656	−10.9
07/40 SR17	UGAMS-5854	charcoal	2980±25	1306–1126	3255–3075	−25.6
07/40 SS22	UGAMS-5857	charcoal	1790±25	134–325 AD	1816–1625	−25.0

* The quotation ‘BP’ refers to uncalibrated years before present, according to Libby's half-life. Calibration using OxCal online version 4.1 [85]

The *corbeilles* differ in their architectural settings, varying from a simple type characterized by a circular perimeter made of slabs vertically set in the ground and often a central standing stone, to more complex structures with external annexes and standing stones. Fillings and stratigraphic settings vary according to the location and the substratum. One or two series of superimposed stones alternating with sand sediments can be set directly over the bedrock or cover a pit dug in the bedrock. The accumulation of faunal remains, including the skull, is usually located at the bottom of the structures.

Stone monuments are not static entities. They were part of a living landscape–sometimes reopened or revitalised. This is evident in some *ab antiquo* ‘plundered’ monuments, such as structures 00/300 and 07/39 C2, and is evident in the rock markings within the monuments and on top of them [25]. *Corbeilles* are not isolated features: stratigraphic relations, analysis of faunal remains and radiocarbon measures helped to better articulate the rituality involved in the use of these monuments. Larger excavations allowed us to reconstruct some of the relations between structures (such as 07/39 C2 and C3; 00/556 and its external stele), where the remains of the slaughtered animals were disposed inside and outside the different monuments.

### Archaeological materials

Although the filling of the structures can occasionally include archaeological finds, firmly associated materials fall in two main categories: stone artefacts (especially maces) and pottery.

**Figure 6:**
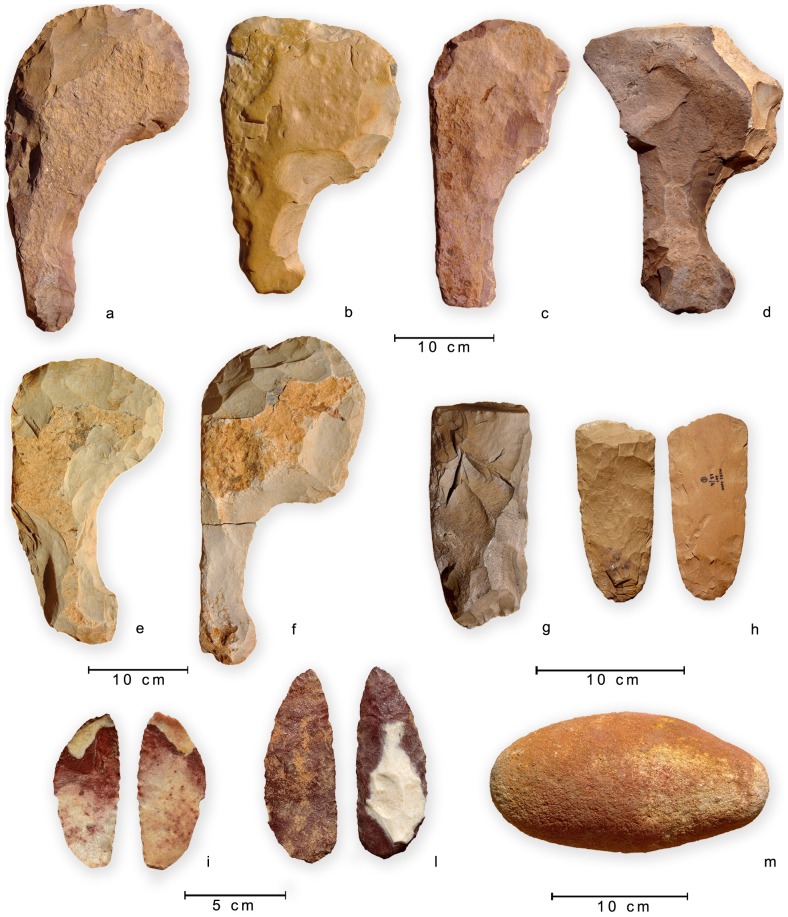
Archaeological materials from the excavation. Selection of stone maces (a–b: 00/301; c–d: 07/39 C3; e–f: 07/110 C1) and other stone tools (gouges, g: 07/110; h: 00/301; scrapers, i–l: 07/79 C1; grinding stone with traces of ochre, m: 07/55 C1).

As a whole (Table S4), we found 16 maces from 11 different structures, mostly *corbeilles*. Maces show similar morphology and opportunistic features (Fig. 6): made on quartzarenite, they are heavy tools (ca. 2.8 kg on average) exclusively produced by means of *façonnage* technique. They can feature a very worn handle, whereas the active protruding part was most likely re-sharpened just before its last use (C. Lemorini unpublished data). They were then ritually placed either in the structure, for example close to the cattle head, or immediately outside the monument. At 07/110 C1, where 3 maces were found, at least one was produced on the very spot, probably to replace a broken one: *façonnage* flakes were placed with the mace close to the animal head and a few could be refitted. Other formal tools, including a grinding stone with traces of ochre (07/55 C1), come from structures 00/301 (1 gouge), 07/79 C1 (2 bifacial knives), 07/110 C1 (1 arrow head, 1 gouge) and 09/69 C1 (1 bifacial tool).

**Figure 7:**
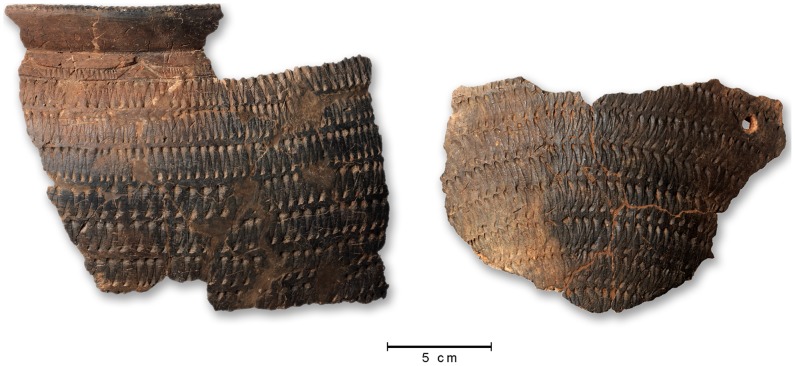
Refitted potsherds from 07/110 C1, showing a rocker plain edge decoration.

Pottery is rare: only 9 structures yielded fragments of pots, usually 1 or 2, with the exception of 00/557 (59) and 00/556 (35), 07/110 C1 (34) and 07/39 C2 (10), for a whole sample of 145 potsherds. Decoration is of the Middle Pastoral tradition, mostly using a rocker stamp/plain edge technique or, less frequently, the Alternately Pivoting Stamp (APS) one (see [65]). Undecorated potsherds are also present. Only at 07/110 C1 the pot could be partially refitted, showing a globular morphology and distinct neck (Fig. 7). In all of the structures but one (07/39 C2), pottery sherds were found in the lowest layers, next to the skull of the animal (when present: 07/39 C3, 07/110 C1, 07/79 C1) or at the bottom of the structure.

To summarize, stone maces represent the authentic emblem of the complex gestures involved in the ritual slaughtering of the animal. Their presence is signalled also in other monumental structures, such as Tin Iblal [62] and most interestingly from the Middle Pastoral quarrying site of In Habeter III [50]. Maces were most likely used to kill the animal(s)–or at least to give them the fatal, symbolic, blow–and their systematic presence in monuments across several centuries supports the high formalization embedded in the ritual. The presence of fragments of pots strengthens the ritual value of the offerings, frequently deposed near the animal skull.

### Analysis of faunal remains

Zooarchaeological analysis provided interesting insights on the rituals performed. Over 25,000 specimens were collected from 30 features belonging to 22 monuments. The distribution of the specimens in the different contexts is variable and only some of them yielded a significant number of identifiable remains (Tables 3 and 4; Text S1; Tables S5 and S6).

**Table 3:** Faunal assemblage.

Structure		Mollusca(Small Gastropoda)	Ostrich (egg shell)	Micromammals	*Equus* sp.		*Ovis vel Capra*		*Bos taurus*		Small Ungulate	Large Ungulate	Indeterminate	TOTAL	% BURNED
		NR	NR	NR	NR	MNI	NR	MNI	NR	MNI	NR	NR	NR	NR	%
00/301	spot A												7	7	100.0
	spot B						71				261		910	1242	57.7
	spot C								3			20	654	677	99.0
	spot D		1				15		34		29	138	2504	2721	97.1
	other	3					1		3		17	8	630	662	95.2
	total	3	1				87	3	40	2	307	166	4705	5309	87.9
00/300				4								3	51	58	15.5
09/69 C1									131	1		95	3803	4029	66.2
07/28 C1									32	1		59	269	360	1.1
07/79 C1									75	1		115	2105	2295	96.9
07/68 C1													83	83	0.0
08/01 C1											1			1	0.0
07/39 C6												1	3	4	75.0
07/39 C5	*corbeille*						1		5	1		7	44	57	10.5
	ext.stele						4						2	9	100.0
	total						5	1	5	1	3	7	46	66	22.7
07/40 SS22													4	4	0
07/10 C1		3											5	8	25.0
07/39 C4	*corbeille*	4					1				14		12	31	58.1
	ext.stele						1				14		62	77	98.7
	total	4					2	1			28		74	108	88.0
07/39 C3									164	2	2	145	3860	4171	99.0
07/37 C1				2									8	10	30.0
07/39 C2	*corbeille*												17	17	0
	ext. area						4	2	49	1	13	105	550	721	80.4
	total						4	2	49	1	13	105	567	738	78.6
07/40 SR13											1		16	17	0
07/40 C1							6	1	159	2	3	93	2864	3125	23.4
07/39 C1					7	1					2	6	36	51	2.0
00/557												2	4	6	33.3
00/556	*corbeille*	1							2		6	18	218	245	81.6
	ext.stele								3		3	36	176	218	91.3
	total	1							5	1	9	54	394	463	86.2
07/110 C1									149	1		178	3813	4140	63.2
07/55 C1									34	1		5	260	299	6.7
Total		11	1	6	7	1	104	8	843	14	369	1034	22970	25345	

(NR = Number of Remains, MNI = Minimum Number of Individuals).

**Table 4:** Synoptic table of the faunal information available for the main structures.

Structure/Feature	Main species	MNI (main species)	Sex	Other species (MNI & age)	Skeleton (main species)	Age teeth	Age post-cranium	Burning	Human modifications	Notes
00/301 spot B	*Ovis vel Capra*	3	?	no	One almost complete, others few fragments.	18–24 m	1:<10–16 m 1: <25–35 m 1: ca. 25–35 m	Total 57.7% Calcined 8.6%	no	see notes 00/301 spot D
00/301 spot C	*Bos taurus*	1	?	no	Very few elements.	n/a	>18 m	Total 99.0% Calcined 98.8%	1 cut mark on a large ungulate rib shaft frag.	see notes 00/301 spot D
00/301 spot D	*Bos taurus*	2	?	*Ovis vel Capra* (MNI = 2; 1 is 18–24 m)	*Bos* one almost complete (only mandible present), other few fragments. Ovicaprine one relatively complete.	18–24 m	2 <36 m	Total 97.1% Calcined 95.1% 96.4% *Bos* & large ung. 73.2% Ovicaprine & small ung. Mandible burnt	2 cuts on indeterm.frgs, 1 scrape mark on a large ungulate rib, 1 chop mark on a small ungulate metapodial.	Considering all the spots together: total Cattle MNI = 2; total Ovicaprine MNI = 3 Parts of these animals may have been distributed in the different spots.
09/69 C1	*Bos taurus*	1	M	no	Quite complete (right front limb absent?); phalanges almost completely missing.	4–6 y (∼4)	2.5–3 y	Total 66.2% Calcined 45.5% Cranium and mandible unburnt.	no	Cranium and mandible still articulated with soft tissues (and skin?) when buried?
07/28 C1	*Bos taurus*	1	?	no	No cranium,only mandible present; distal limb elements missing.	8–10 y	∼5 y	Total 1.1% No calcined. Mandible unburnt.	Fresh bone fractures on a cattle humerus and on a large ungulate long bone shaft frag.	
07/79 C1	*Bos taurus*	1	?	no	Largely incomplete.	4–6 y (∼6)	3.5–4.5 y	Total 96.9% Calcined 13.0% Mandible burnt, cranium unburnt.	Disarticulation cut mark on a large ungulate rib.	High frequency of burning damage, but less intense (relatively few calcined specimens) than other structures.
07/39 C5 *corbeille* & ext.stele	*Bos taurus (corbeille)/Ovis vel Capra* (stele)	1 *Bos* + 1 Ovicaprine	?	n/a	Very few elements for both species.	n/a	"adults"	Total 22.7% Calcined 15.2 *Corbeille* 10.5% Stele 100%	Cut mark on a small ungulate long bone shaft frg.	
07/39 C4 *corbeille* & ext.stele	*Ovis vel Capra*	1	?	no	Few elements, but from different parts of the skeleton.	n/a	18–26 m	Total 88.0% Calcined 75.7% *Corbeille* 58.1% Stele 98.7%	no	Same individual deposited between *corbeille* and ext. stele?
07/39 C3	*Bos taurus*	1(+1?)	M	Small ungulate 2 frgs.	Almost complete;humerus missing; a single extra metacarpal indicates second individual.	4–6 y (∼6)	2.5–3 y	Total 99.0% Calcined 98.4% Mandible burnt, cranium unburnt	no	The extra metacarpal of this individual may belong to the 07/39 C2 ext.area cattle.
07/39 C2 ext.area	*Bos taurus*	1	?	*Ovis vel Capra* (MNI = 2; 3 humeri, "adult")	Almost complete (left front limb absent?) mandible missing, but lower teeth present.	4–6 y (∼5)	2.5–3.5 y	Total 80.4% Calcined 60.6% Cranium and upper teeth burnt; lower teeth unburnt.	Two worked small ung. long bone shaft frgs.; a bone spatula on small ung. rib. Two large ung. long bone shaft frgs. with impact cone. Large ung. scapula blade with defleshing cut marks.	
07/40 C1	*Bos taurus*	2	1 M	*Ovis vel Capra* (MNI = 1;18–24 m; few specimens)	One almost complete; only few elements of the front limb and a tooth indicate the presence of the second individual.	7–9 y	4.5–5 y	Total 23.4% Calcined 0.03% Cranium and mandible burnt	Disarticulation cut marks on two ribs, a scapula and a pelvis of cattle.	
07/39 C1	*Equus* sp.	1	?	Small ungulate 2 frgs.	Almost only upper teeth and mandible.	"adult"	n/a	Total 2.0% (all calcined) Only a vertebra frg. burnt. Head elements unburnt.	no	
00/556 *corbeille* & ext.stele	*Bos taurus*	1	?	Small ungulate 9 frgs.	Largely incomplete; cranium, mandible and teeth in platform;post-cranium mainly in stele.	n/a	>24 m	Total 86.2% Calcined 84.4%*Corbeille* 81.6% Stele 91.3% Cranium burnt, mandible unburnt.	no	Same individual deposited between *corbeille* and ext. stele?
07/110 C1	*Bos taurus*	1	M	no	Almost complete; lower teeth missing, but mandible present.	4–6 y (∼5)	3–4 y	Total 63.2% Calcined 40.7% Mandible burnt, cranium unburnt.	Skinning traces on a metatarsal and disarticulation traces on a rib, both of cattle.	
07/55 C1	*Bos taurus*	1	?	no	Almost only cranium and mandible	4–6 y (∼6)	n/a	Total 6.7% Calcined 1.3% Mandible and cranium unburnt.	no	Low incidence of burning may be explained by the scarcity of postcranial elements. Cranium and mandible still articulated with soft tissues (and skin?) when buried?

As far as species are concerned, 17 features contain cattle or large ungulates: in 6 cases *Bos taurus* was the only species recovered, in 3 others large ungulate was the only taxonomic category, while in 8 occurrences cattle is associated with ovicaprines or small ungulates. In these latter cases, however, small livestock is usually represented by few fragments, the only exception is structure 00/301 where *Ovis* vel *Capra* specimens are more abundant. Ovicaprines or small ungulate were the only taxon identified in 6 features. Structure 07/39 C1 yielded some equid specimens associated with very few small ungulates. In the last 5 features only unidentifiable fragments were recovered.

In most structures, for the main species a single individual is present (cattle, caprine or equid). The exceptions, with two *Bos*, are 07/40 C1 and 07/39 C3: in the latter, however, the single extra fragment may belong to the animal of 07/39 C2 external area, likely connecting the two monuments. Structure 00/301 with a minimum number of 2 cattle and 3 ovicaprine individuals represents an extreme outlier: here there seems to be also a differential distribution of cattle and small livestock in the various points of fire identified during the excavations. A similar differential distribution of species was found in 07/39 C5 where cattle remains were collected only in the *corbeille* and ovicaprines mainly under the external stele.

In most cases, the poor state of preservation of the bones prevented further assessments (sex, short-horn vs. long-horn, etc.) and only a few specimens could be measured (Table S5). Comparisons with available metric data from other sites in North Africa e.g., [26], [66] show that the animals from the Messak were of similar size or slightly larger. The sex of *Bos taurus*, based of the size and morphology of metapodials, was tentatively attributed only for 4 animals, all males (Table 4): one of them is from 07/110 C1 where the close rock art engraving (Fig. 2) shows the sacrifice of a bull. A further example of this sexual selection is represented by the bull from In Habeter III [63].

The analysis of body part frequencies was achievable only for 18 features (Table S6). Cattle shows some variability: the skeleton is almost complete in 6 contexts; in five other cases the head (cranium and/or mandible) is preserved, sometimes associated with only few other elements. At 00/556 there seems to be a patterned distribution of the skeletal elements with the head placed in the *corbeille* and the long bones found at the basis of the external stele. Radiocarbon dates of the two samples, even if slightly different, have overlapping sigmas (Table 2). The cranial portions of the animal seem to have been important also at 07/39 C1 where the equid specimens were recovered. The anatomical pattern for the ovicaprines is usually less complete and standardized, except for structure 00/301 where almost all the skeleton is present. The only possible evidence for a selection of ovicaprine elements may occur at 07/39 C2 external area where 3 out of 4 identified specimens are humeri.

Only in a few cases it was possible to indicate the age at death of the animals (Table 4; see Text S1 for discussion). Except for structure 00/301 where cattle are less than 36 months old, the bovines are mainly adults, while the few ovicaprines tend to be younger. All the other individuals of the identified species could only generically be considered as “adults”.

Given the poor state of preservation of the assemblage, it was difficult to observe bone surface modifications. As a consequence, butchering traces are apparently very rare, but related to different stages of carcass processing, from skinning, disarticulation and defleshing to bone fracturing for marrow extraction.

A large proportion of the fragments was burnt: the incidence of fire damage on the bones is usually very high, often with many calcined specimens.

Differences were also observed in the location of these traces, in particular among the head portions. In structures 07/55 C1 and 09/69 C1 the presence of unburnt cranium, mandible as well as hyoid fragments may suggest that the cattle head was placed in the structure with soft tissues still attached. Differences in the frequency of burning were recorded also between cattle and ovicaprines when present in the same structure in significant numbers, as well as sometimes between the *corbeille* and the associated external stele.

Considering the available faunal data it is clear that, although with some variability and few exceptions, the ritual in this region was quite standardized. In most contexts domestic cattle played the main role with ovicaprines representing only a secondary species, as also suggested by the different treatment of the two animals (e.g., frequency of specimens, skeletal element representation, age, burning). The only real outlier is structure 00/301 where the rituals seem to involve in a similar manner *Bos taurus* and *Ovis* vel *Capra*, however such anomaly could be explained by the fact that this is the oldest structure analysed.

Age selection indicates that for cattle mainly adult individuals were chosen, while for the ovicaprines younger animals were preferentially killed. In other African sites [26], [30], age data indicate slightly less mature animals. In the Messak, probably only bulls, rather than cows as in the case of some Egyptian and Niger sites, were selected for the sacrifice. The head of the animal was considered a relevant portion and was often placed at the bottom of the structure while, at least in some cases, the rest of the carcass was skinned, disarticulated and meat as well as marrow were consumed before the “leftovers” were collected and deposited in the monument after being intentionally burnt. Such intentionality is suggested in many contexts by the high frequency of calcined bones, which cannot be merely the result of cooking processes. On the basis of age and sex of the cattle, a large amount of meat was available (with the addition in some cases of the ovicaprines), suggesting that many people took part to the ritual. In a few structures (e.g., 00/301; 07/39 C2 and C3; 07/40 C1) more animals were slaughtered; this could be the evidence of special gathering places.

In other North African ritual sites with cattle bones the animal or parts of it are usually still articulated and burning is not a common occurrence e.g., [26], [30], [67]. Some similarities may be found with the so called “Tenerian meals” found in the Adrar Bous area [31], especially for the high incidence of burning, mainly on cattle elements, produced after consumption. However, there are dissimilarities in secondary species composition, number of individuals, anatomical representation as well as archaeological context.

The type of ritual identified in the Messak, although involving the same species of other North African areas, shows marked differences in the age and sex of the animals, as well as carcass treatment; they reflect the existence of a regional tradition, which given its level of standardization might have lasted over several centuries.

### Isotope study

To have a measure of the environmental conditions in the area during the Pastoral phase and explore cultural phenomena linked to animal mobility we performed an isotope investigation on the animals buried in the stone monuments in the Messak and nearby areas. Animal stable isotope history was explored at the seasonal scale by means of sequential sampling of enamel along the tooth crown axis for carbon (δ^13^C) and oxygen (δ^18^O) isotope analysis [68], while local/exogenous origin and seasonal mobility were investigated through strontium isotope ratio (^87^Sr/^86^Sr) measured at the two furthermost oxygen values.

The state of preservation of the faunal remains was generally rather poor, we thus decided to sample only teeth in good conditions; out of 17, twelve came from the Messak (MK, respectively 10 of *Bos taurus*; 1 of *Equus* sp.; 1 of *Ovis* vel *Capra*), while 4 teeth were selected from the Edeyen of Murzuq (MQ, respectively 2 of *Bos taurus*; 1 of *Ovis* vel *Capra*; 1 of *Hippopotamus amphibius*), and 1 from the Erg Uan Kasa (UK, *Bos taurus*).

For strontium isotope analysis we collected 8 further specimens of terrestrial shells (*Pupoides hogarensis*) from the stone structures in the Messak: bulk readings could provide a measure of the local Sr isotope signature. While terrestrial shells are good indicators of the local geology, their association with the structures remains uncertain thus data should be considered with caution. Three samples of carbonate concretions from the nearby wadis were also collected. Bulk analysis of modern animal teeth from other areas (respectively 3 modern goat teeth–leftovers provided by local–from the area of Mathendous in the Messak and 2 teeth of *Ammotragus lervia* from carcasses found during our surveys in the Acacus Mountains) was performed as a further proxy of the local geology (see Fig. 1).

We excluded from our sampling burnt materials. The general poor state of preservation of the teeth only allowed the subsampling–for C and O isotope analysis–of 11 individuals from the Messak area (respectively 9 of *Bos taurus*, 1 of *Equus* sp. and 1 of *Ovis* vel *Capra*) and 2 from the Murzuq area (2 *Bos taurus*).

A general background of C, O and Sr isotope studies, together with methods of pre-treatment and analysis are included with the supplementary material (Text S1).

Only 9 teeth sequentially sampled for O and C isotope analysis yielded reliable results (respectively 7 *Bos taurus* and 1 *Equus* sp. from the Messak area and 1 *Bos taurus* from the Murzuq–the ovicaprines, mostly of young age, did not preserve enough enamel) (Table 5).

**Table 5:** Carbon (δ^13^C), oxygen (δ^18^O) and strontium (^87^Sr/^86^Sr) isotope ratio of tooth enamel bioapatite, carbonate rock and terrestrial shells from archaeological and modern specimens collected in the Libyan Sahara.

Area code*	Sample ID	species/material	tooth	sub-sample**	Mm from cej***	δ^13^C-VPDB	δ^18^O-VPDB	^87^Sr/^86^Sr	% standard error
MK	07/28 C1	*Bos taurus*	M2 lower	1	18	−2.0	0.7		
				2	16	−0.7	0.2		
				3	12	0.0	0.1		
				4	10	−0.5	0.6		
				5	8	−0.6	0.6		
				6	4	0.3	−0.2		
MK	07/79 C1	*Bos taurus*	M3 upper	1	28	1.5	2.7		
				2	25	1.8	3.2	0.709748	0.00070
				3	21	2.3	2.1		
				4	17	2.4	1.7		
				5	13	2.2	0.9		
				6	10	2.3	0.8	0.709717	0.00080
MK	07/39 C3	*Bos taurus*	P2 upper	1	21	2.1	4.4		
				2	19	2.4	4.2		
				3	17	2.4	4.1		
				4	15	2.5	4.3	0.709815	0.00060
				5	13	2.3	3.6		
				6	11	1.9	2.9		
				7	9	1.5	2.2	0.709814	0.00080
				8	7	1.1	2.9		
				9	4	0.6	3.0		
				10	2	0.3	3.9		
MK	07/39 C2 ext.	*Bos taurus*	M3 lower	1	33	−5.9	−1.0	0.709715	0.00090
				2	29	−1.5	2.1	0.709839	0.00070
				3	26	−1.2	1.9		
				4	23	0.1	2.1		
				5	20	1.0	1.9		
				6	17	2.4	1.6		
				7	14	2.8	1.4		
				8	11	2.7	0.6		
				9	7	2.7	1.4		
				10	3	3.4	0.6		
									
MK	07/40 C1(A)	*Bos taurus*	M1 upper	1	8	2.2	0.9	0.709775	0.00070
				2	6	2.5	1.5		
				3	4	2.3	1.7	0.709779	0.00070
MK	07/40 C1(B)	*Bos taurus*	M1 upper	1	5	−6.7	4.0	0.709721	0.00060
MK	07/39 C1	*Equus sp.*	M3 upper	1		−1.4	2.8	0.708718	0.00070
				9		−0.9	0.8	0.709706	0.00070
				10		−0.5	1.1		
				11		−2.1	1.0		
				12		−3.3	1.5		
				13		−3.3	0.9		
				14		−2.7	1.2		
MK	00/556 *corbeille*	*Bos taurus*	fragment (M?)					0.709981	0.00080
MK	00/556 *corbeille*	Small ungulate cf ovc	fragment					0.709671	0.00080
									
MK	07/110 C1	*Bos taurus*	M3 upper	1	36	−2.7	1.0		
				2	34	−3.4	1.9		
				3	31	−3.4	2.2		
				4	28	−3.6	2.5		
				5	26	−3.2	2.6		
				6	23	−2.7	2.4		
				7	20	−1.7	2.7	0.709852	0.00080
				8	18	−0.8	2.4		
				9	14	0.3	1.7		
				10	10	0.1	1.3		
				11	6	−0.1	0.1	0.709867	0.00070
MQ	M4A/34	*Bos taurus*	M2 upper	2		−3.5	0.8	0.709975	0.00070
				3		−3.7	1.1		
				4		−2.6	1.5		
				5		−2.5	1.9		
				6		−2.5	2.2		
				7		−1.4	2.2	0.709967	0.00060
				8		0.1	1.2		
				9		0.8	1.9		
				10		0.8	1.0		
									
MQ	M4/226	*Hippopotamus amph.*	fragments					0.709812	0.00070
MQ	MT136	*Ovis vel Capra*	P2					0.709824	0.00080
									
MQ	M4A/166	*Bos taurus*	M2	1				0.709873	0.00060
				7				0.709853	0.00080
UK	94/75	*Bos taurus*	fragments (M?)					0.711068	0.00070
MK	#1 Wadi Bedis	*Carbonate concretion*						0.709538	0.00070
MK	#2 Wadi Mathendous	*Carbonate concretion*						0.709693	0.00080
MK	#3 Wadi Tullult	*Carbonate concretion*						0.709711	0.00070
									
AC	Ammo 1	*Ammotragus lervia*	M2 lower					0.710710	0.00070
AC	Ammo 2	*Ammotragus lervia*	M2 lower					0.710656	0.00070
	Libyan Modern Goat 1	*Capra hircus*	M2 lower					0.710079	0.00080
	Libyan Modern Goat 2	*Capra hircus*	M2 lower					0.710171	0.00070
	Libyan Modern Goat 3	*Capra hircus*	M2 lower					0.709880	0.00070
MK	07/10 C1	*Pupoides hogarensis*						0.709701	0.00080
MK	07/10 C1	*Pupoides hogarensis*						0.709769	0.00080
MK	07/10 C1	*Pupoides hogarensis*						0.709764	0.00080
MK	07/39	*Pupoides hogarensis*						0.709675	0.00070
MK	07/28	*Pupoides hogarensis*						0.709707	0.00070
MK	07/28	*Pupoides hogarensis*						0.709612	0.00070
MK	07/110	*Pupoides hogarensis*						0.709680	0.00070
MK	00/556	*Pupoides hogarensis*						0.709623	0.00070

* Area code: MK: Messak; MQ: Murzuq; AC: Tadrart Acacus; UK: Uan Kasa (see Fig. 1): ** Sub-sample no. refers to the sequential sampling of enamel along the tooth crown; *** abbreviations: cej = cement-enamel junction; M = molar; P = premolar.

**Figure 8:**
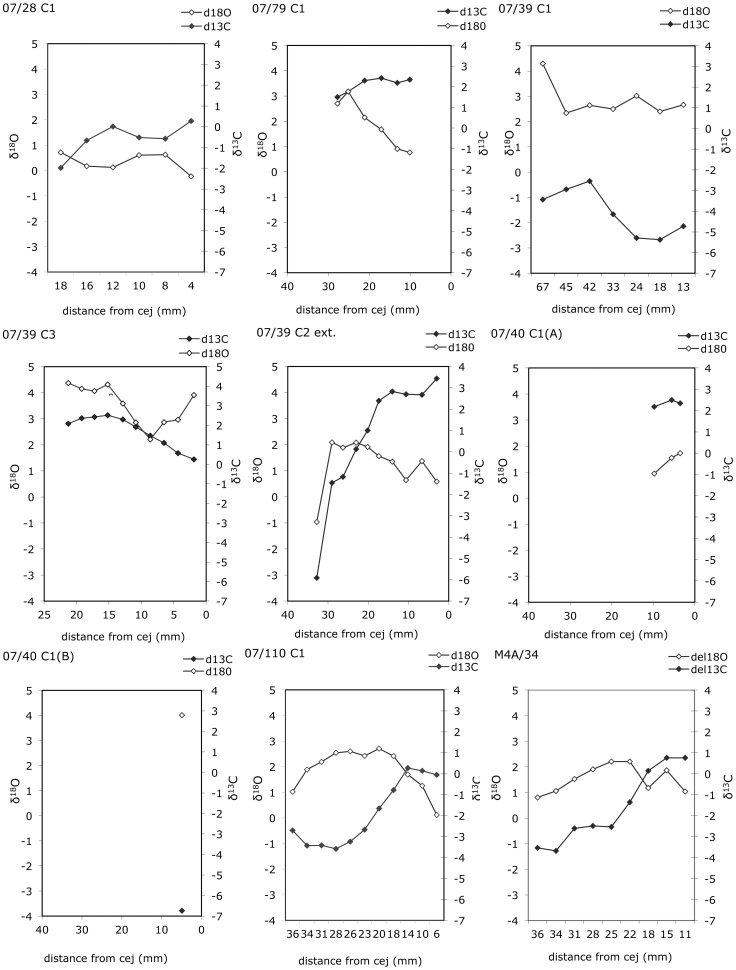
Carbon (δ^13^C) and oxygen (δ^18^O) data. Intra-tooth variation of carbon (solid diamonds) and oxygen (open diamonds) isotope ratios (in ‰) of enamel bioapatite of archaeological animals from the Messak and Murzuq. Abbreviations: cej = cement-enamel junction.

The δ^13^C values vary from −6.7 to 3.4‰ in bovine molars and premolars and from −3.3 to −0.5‰ in the equid molar. Excluding the very short sequences measured in 07/40 C1 individuals A and B, intra-tooth variability varies from 0.9‰ to 9.3‰ for δ^13^C and from 1.4 to 3.1‰ for δ^18^O in bovine teeth (Fig. 8). In the equid molar, intra-tooth variability is 2.8‰ for δ^13^C values and 2‰ for δ^18^O values. Within each sequence, the highest δ^13^C values occur shortly after the δ^18^O values reach their maximum, in agreement with what would be expected from the natural vegetation cycle, with a higher relative abundance of C_4_ plants and/or higher δ^13^C values for C_3_ plants in the dry season, and/or higher δ^13^C values for C_3_ plants in the wet one [69]. At these latitudes, with temperatures exceeding the amount effect threshold [70], the variations recorded in the δ^18^O values reflect seasonal variation in precipitation, with the highest δ^18^O values reflecting the dry season (winter), while in most sampled teeth, the wet (summer) season is truncated from the sequence, biasing the δ^13^C values recorded in tooth enamel towards dry season diet. The stable isotope sequences measured in the four bovine molars (07/39 C2 ext. area; 07/110 C1, 07/79 C1 and M4A/34) show very similar trends, with the highest δ^18^O and δ^13^C values occurring approximately at a distance from the enamel-root junction of 25 mm in the first case and 15 mm in the further three.

^87^Sr/^86^Sr ratios from the Messak area range between 0.70966 and 0.70998 for the bovine teeth, and 0.70971 and 0.70972 for the *Equus*, while the only sheep/goat sample has a signature of 0.70976. The bovine specimens from the area of Murzuq range between 0.70985 and 0.70998, with 0.70982 for the sheep/goat sample and 0.70981 for the hippopotamus. Hence, there is a substantial overlap in the Sr isotope signature from samples of the two areas. Significantly, the bovine specimen from the Erg Uan Kasa represents the only exception, with a Sr isotope signature of 0.71107 (Table 5). Mean Sr ratio of the terrestrial shells is 0.70968±0.00006, while the carbonate samples range between 0.70954 and 0.70971. Modern Sr isotope signature is not dissimilar to that of the prehistoric specimens: the two wild ruminants have comparable values that average 0.71068 while the goats range between 0.70988 and 0.71017.

**Figure 9:**
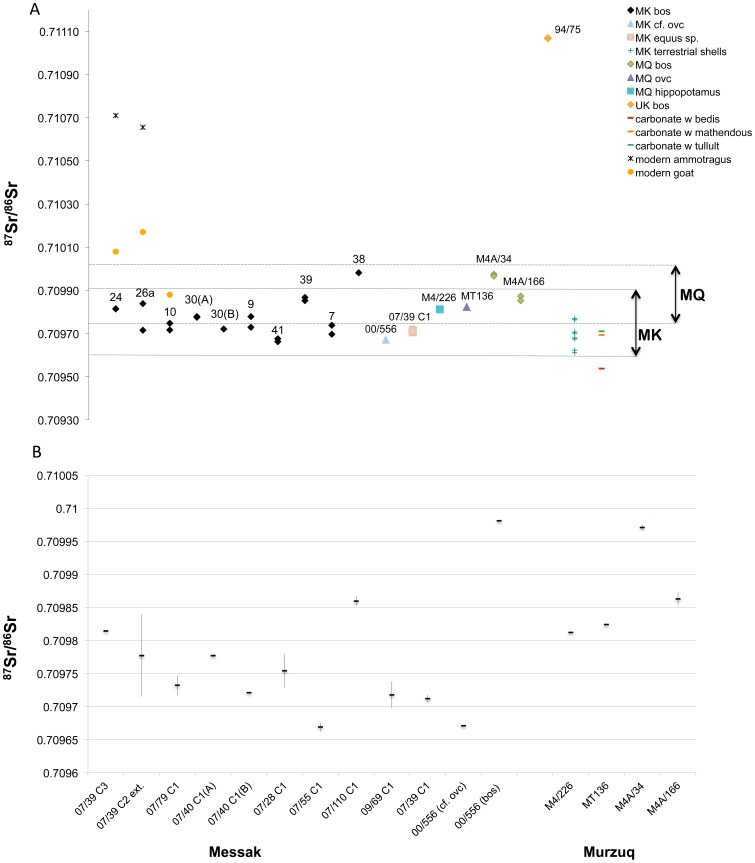
Strontium isotope ratio (^87^Sr/^86^Sr) of archaeological and modern samples. (A) For both the Messak (dotted line) and Murzuq (dashed line) the local range is defined by 2 sd of the enamel mean values of the ancient animal samples. Intra-individual Sr signatures are measured at the two furthermost oxygen values. Messak sample codes: 24 = 07/39 C3; 26a = 07/39 C2 ext.; 10 = 07/79 C1; 30(A) = 07/40 C1(A); 30(B) = 07/40 C1(B); 9 = 07/28 C1; 41 = 07/55 C1; 39 = 07/110 C1; 7 = 09/69 C1; 38 = 00/556. (B) Range of strontium isotope ratio (^87^Sr/^86^Sr) for the Messak and Murzuq animals. Abbreviations: MK = Messak; MQ = Murzuq; UK = Uan Kasa.

All animals from the Messak monuments appear to be feeding on similar geological substrates. When defining the local range using 2 times the standard deviation of the mean Sr isotope values of the ancient enamel samples [71], all cattle show a common ‘local’ origin (Fig. 9a). However, four individuals (07/39 C3, 07/39 C2 ext. area, 07/110 and 00/556) despite being local, cluster on the furthermost values of the local mean and group with the specimens from the Murzuq (Fig. 9a). The bovine sample from the Erg Uan Kasa falls outside both Messak and Murzuq ranges, which is unsurprising given the geological background of the area; the Sr signature of this single specimen appears more in line with those of humans of different Pastoral ages from the Wadi Tanezzuft and Wadi Takarkori [18], [39]. The signatures of modern goat samples are consistent or close to the geological background of the sample area even if, in two cases, they might reflect the contribution of imported fodder to the diet of these animals. The *Ammotragus* samples confirm an origin non-local to the Messak or Murzuq, and match that of the area of sampling (Acacus Mts.).

When combining δ^13^C and δ^18^O data with Sr isotope ratio, the picture becomes more integrated.

Similar trends in the sequences of δ^18^O and δ^13^C values in 07/39 C2 ext. area; 07/110 C1, 07/79 C1 and M4A/34 suggest these individuals were born at the same period of the year. Given that in extensive conditions, cattle breeding cycle is driven by environmental variables including the vegetation annual cycle [72], this would suggest that these individuals were born in areas similar at least from this point of view. Still, a great inter-individual variability in the range of δ^13^C values suggest they grazed on different pastural areas. The latter seems to be confirmed, at least for individual 07/39 C2 ext. area and 07/79 C1, by the range of the Sr isotope signature at the furthermost peaks of δ^18^O (dry vs. wet season) which appears to indicate that the bovines have moved between two geologically different areas (Fig. 9b). Unfortunately individual 09/69, which also suggests mobility, yielded unreliable C and O data.

Using a 14.1 ‰ isotope enrichment (ε*) of ^13^C between diet and enamel bioapatite [73], the δ^13^C values measured in the enamel were converted to diet δ^13^C values, leading to an estimation of the relative proportion of C_3_ and C_4_ plants in diet, using the mean values of −25.5‰ and −11‰ for pre-industrial C_3_ and C_4_ plants (see Text S1). Individuals may be grouped according to the relative proportion of C_4_ plants in their diet. 07/40 C1 individual B, although represented only by one value, is the only tooth that gave a δ^13^C value reflecting a C_3_ dominated signal (approximately 60% C_3_ in diet). In 07/39 C3, 07/79 C1, and 07/40 C1 individual A (represented only by three values), C_4_ plants are largely dominant (≥90%) to exclusive in diet. These δ^13^C values are comparable to those measured in bovine teeth from low altitude modern and Neolithic (Elmenteitan) pastoral settlements in the savannah grassland of the Central Rift Valley in Kenya [74]. In 07/110 C1, M4A/34 and 07/28 C1, C_4_ plants dominate in diet, but a fair contribution of C_3_ plants is also detected seasonally (approximately 30–40%). This group of individuals gave similar range of δ^13^C values as the *Equus*. They may also be compared to δ^13^C values measured in cattle tooth enamel from historical and Elmenteitan occupations at higher elevation (2600 m) in the Central Rift Valley in Kenya, for which altitudinal mobility is suspected [74]. 07/39 C2 external area has the widest range of intra-tooth variation for δ^13^C values (9.3‰). In this tooth were measured the highest δ^13^C values of the sample (3.4‰) but also one of the lowest δ^13^C values (−5.9‰) suggesting a contribution of approximately 54% C_3_ plants to diet seasonally, this is also one of the individuals with greater variation in the Sr signal; the two proxies might suggest mobility between two diverse environments.

This great variability in the relative proportion of C_3_/C_4_ plants in the bovine diet is higher than what could be expected from individuals grazing in a single location and may suggest that these animals were coming from different places. Variability is also indicated by residue studies on potsherds from sites in the nearby Acacus Mountains [7]. We do not expect mean δ^13^C and δ^18^O values to be correlated in this sense (they are not), but would rather explain these different signals as reflecting diverse herding practices in terms of grazing areas, including location of pastures in altitude and possible seasonal mobility during the year to cope with variations in rainfall and other environmental constraints [34], [35], [38].

The Sr signature from all of the individuals (either Messak and Murzuq) is coherent with such a scenario, ^87^Sr/^86^Sr in most of the animals suggests a common origin. Within the Messak sub-sample, the 4 outliers fall within the Murzuq range. The integration of δ^13^C and δ^18^O data with the ^87^Sr/^86^Sr ratio outlines a picture of ‘local’ animals, mostly grazing on C_4_ plants though accessing diversified pastures, likely in connection to intra-annual mobility between geologically consistent areas (along the wadis of the Messak and in the dunes of the Edeyen of Murzuq).

The integration of δ^13^C, δ^18^O and ^87^Sr/^86^Sr data is particularly interesting for two structures (07/39 C3 with C2 external area and 07/40 C1). The two bovines in structure 07/39, even if hypothetically born at different times of the annual cycle show very different values, especially in the δ^13^C, so as to suggest different pastural areas. The discrepancy in the intra-individual mobility of the two animals, as reflected in the Sr isotope ratio supports this scenario.

Structure 07/40 C1 also contained two bovines, which were local as far as Sr isotope ratio is concerned, but–despite having both very short δ^13^C and δ^18^O sequences, show rather different values hence different proportions of C_3_ and C_4_ plant contribution to their diet.

In both such cases a single structure or a single architectural context, host animals likely to thrive on different pastures or move at different scales of resolution. It is tempting to suggest that these animals might have been parts of different herds, which conveyed at a same area or were part of a same ritual.

### Archaeobotanical analyses

To investigate possible plant accumulation in burials, a random set of botanical samples was taken from four well preserved monuments with *Bos taurus* bones, together with a few preserved and naked-eye visible remains of plants.

**Figure 10:**
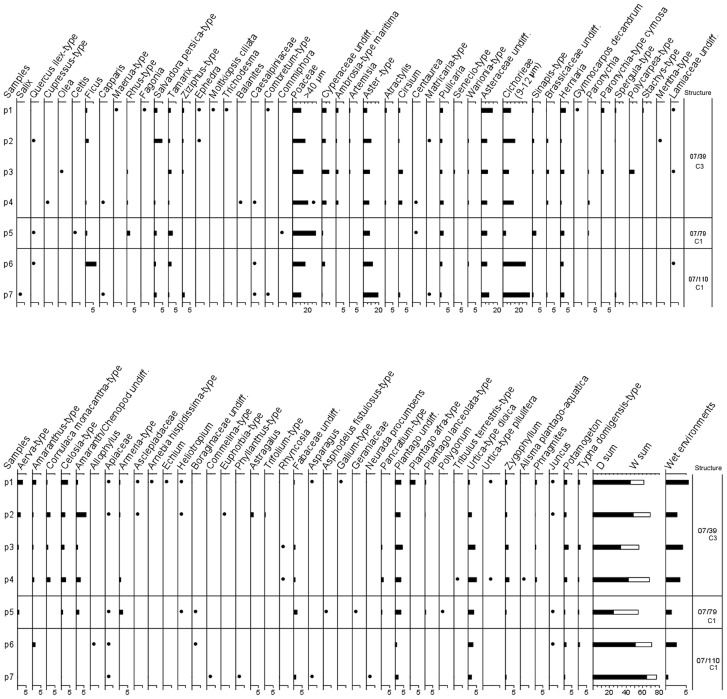
Percentage pollen diagram of three structures showing most of the identified pollen types. Selected pollen sums (bottom) include the D (dry) and W (wet) sums, and pollen from plants living in water habitats.

Pollen samples (structures 07/39 C3, 07/79 C1, 07/110 C1) were treated according to [75], and macroremains (structures 07/79 C1, 07/110 C1, 09/69 C1) were sorted under stereomicroscope (Text S1). The main results are reported in Table 6. Pollen flora is fairly similar in the different structures, showing prevalence of non arboreal pollen (NAP) and presence of tropical taxa (Fig. 10). Pollen spectra are dominated by the daisy family-Asteraceae (38% on average, 12 pollen types besides the undifferentiated Asteroideae) and by grass family-Poaceae (17%). Chenopods belonging to Chenopodiaceae/Amaranthaceae (6%), herbs of the carnation family-Caryophyllaceae (5%) and sedges-Cyperaceae (4%) are less represented. Plantains-*Plantago* and nettle family-Urticaceae are 3% each. Trees are low (7% on average) confirming that vegetation was open, and only fig tree-*Ficus*, toothbrush tree-*Salvadora persica* and tamarisks-*Tamarix* reach 1–1.5% on average. The sums of Asteraceae+Chenopodiaceae/Amaranthaceae (D) and Poaceae+Cyperaceae (W) indicate that the dry shrubland is almost always more represented than the savannah vegetation in the spectra. The D/W ratio is <1 only in sample p5 (structure 07/79 C1) as a result of the local abundance of grass pollen.

**Table 6:** Botanical samples and main results of microscopical analyses of pollen and plant macroremains.

Structure	Age uncal. BP	Layer	Sample*	Sample location	Depth (cm)	Pollen concentration (p/g)	Results of analyses of pollen and macroremains**
07/39 C3	5520±30	3	p 1	inner area	38	2600	see description in 07/39 C3 sample p 4
		4	p 2		43	1500	see description in 07/39 C3 sample p 4
		5	p 3		45	3100	see description in 07/39 C3 sample p 4
		6	p 4		47	1000	Pollen from dry habitats, including 33% of Asteraceae, is high. Psammophilous habitats with *Cornulaca*, *Ephedra* and *Moltkiopsis*, which were not found in the other sites, are present. Plants from wet environments (4.0%) and *Armeria* (0.4%) are represented too. Tropical trees are represented by the Sudanian *Balanites* and Caesalpiniaceae (in p4), and the Sahelian *Maerua* (in p1)
07/79 C1	5400±30	3	mc 3	below inner ring	27		*Rumex cyprius/vesicarius* (n. 400)
		2	mc 2	external ring	30		*Rumex cyprius/vesicarius* (174 fruits); *Ficus* (19 fruits); *Picris* (9 fruits); *Echium* (1 fruit); Poaceae (10 stems and fragments; one floret of *Pennisetum* sp.); pods of Fabaceae (10); small flowers of Boraginaceae (9) and thorns (4)
		2	mc 1	inner area	33		*Rumex cyprius/vesicarius* (250 fruits); *Ficus* (5 fruits); Poaceae (fragments)
		7	p 5	inner area	44	3500	The lowest D/W sum is observed in this sample, especially because Asteraceae are 21%. Besides fresh-water habitats (1.4%), places with brackish water hosting *Armeria* (1.9%), and tamarisks on shores, are represented. The tropical-Sahelian *Commiphora* is exclusive.
07/110 C1	5380±25	—	p 6	eastern wall	184	830	see description in 07/110 C1 sample p 7
		—	p 7	eastern wall	214	730	Asteraceae, covering 57% of the spectra, testifies that drought-resistant shrubs and herbs grew near the site. The high value of *Ficus* observed in p6 (6.5%) is a local over-representation. *Cassia* and *Combretum* are example of the tropical taxa present in these deposits. Some evidence of local wet conditions is recognisable because the aquatic *Potamogeton* (0.9% on average) needs permanent water, here probably small mires, and the xerophilous chenopods were not found.
		5	mc 4	stele pit	145		*Rumex cyprius/vesicarius* (n. 46)
09/69 C1	5350±25	6	mc 5	pit			few and small fragments of charred twigs and stems

* p = pollen; mc = macroremains; ** Seeds/fruits are reported as number of records counted.

**Figure 11:**
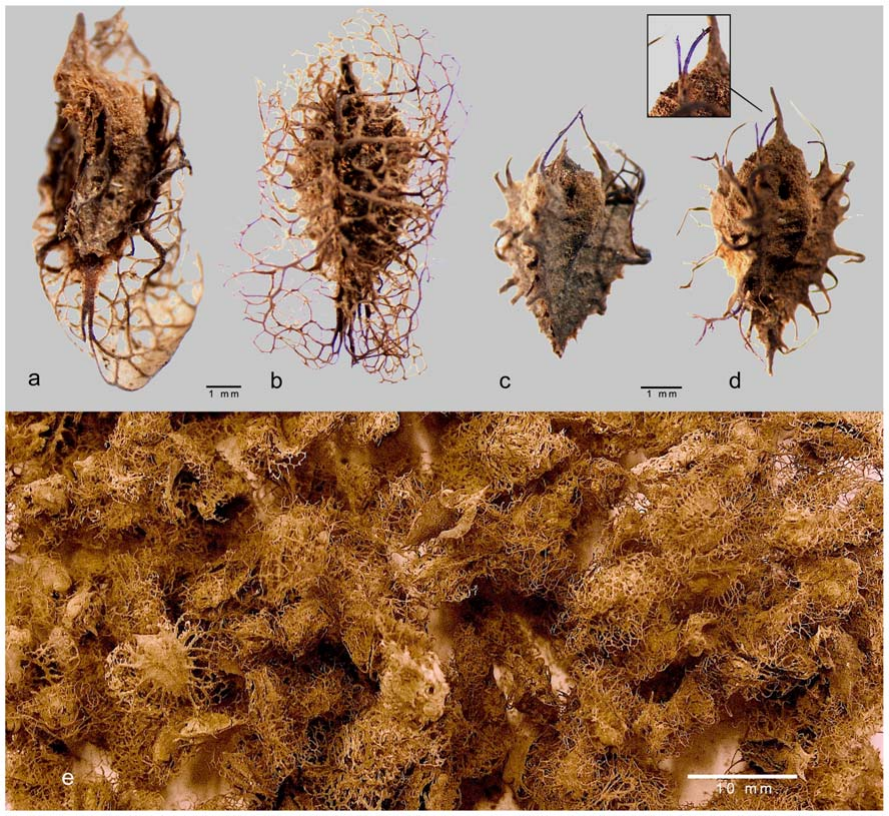
Botanical remains. Fruits of *Rumex cyprius/vesicarius* from structures 07/79 C1 (a, c = sample mc1; b, e = sample mc3) and 07/110 C1 (d = sample mc4). Record a still preserves the membranaceous parts of one wing; b has the remains of the wings, while records c and d lost their wings; d has still some purple colours in the remains of the veins (see detail).

Seeds and fruits are well preserved in a desiccate state. Remains mainly consist of fruits of *Rumex cyprius/vesicarius* (cypriot dock/sorrel; Fig. 11) that amounts to 93% of the carpological record. Whole or fragmented fruits are preserved together with fruits (achenes) of *Ficus* and stem fragments of Poaceae in sample mc1, while other types of records are present in sample mc2. In structure 09/69 C1, only a few charred stems and twigs were found, while fruits were absent (Table 6).

Altogether, data show that the environments near the monuments were characterised by desert shrublands that periodically became brackish alternated with fresh-water habitats (especially at structures 07/79 C1 and 07/110 C1). Interestingly, the pollen list includes a significant number of tropical tree taxa (e.g., *Balanites*, *Commiphora*, *Salvadora*) spread in the Sahara during the mid-Holocene [76], [77].

Inside structure 07/110 C1, a low concentration of microscopical organic matter, including that of pollen and charcoal particles, was observed. The sum of *Plantago*+*Urtica* pollen is 3.3%, and this is an indication of some trampling and frequentation around the monument [78]. A significant amount of *Rumex cyprius/vesicarius* was found in sample mc4, collected at 145 cm depth: given the exclusive presence within this level, we interpret this as evidence of an intentional deposition inside the burial.

Samples from the other two monuments, 07/39 C3 and 07/79 C1, contain more organic matter, probably related to a higher local accumulation of plants as flowers, fruits and charcoals. A relatively more intense frequentation around these two structures is signalled by the *Plantago*+*Urtica* sum (8.0–7.4%, respectively). In structure 07/79 C1, sample mc3 contains a very high amount of *Rumex cyprius/vesicarius* and, just below, sample mc2 shows the highest diversity of seeds/fruits in these deposits. In this monument, a special consideration for this plant is confirmed, though also other plants were collected.

On the basis of these data some palaeo-ethnobotanical inferences can be drawn. Burials have specific features that make their archaeobotany fairly different from that of settlements. Sometimes, distinctive pollen assemblages witness floral depositions and rituals, and mixed pollen spectra could reveal subsequent input of terrigenous material in burials e.g., [79]. According to [80], these contexts are particularly hard to interpret since samples can include pollen and plant remains from different sources (floor context, objects lain on the floor, stomachs, hairs, etc.). As contamination may have occurred in several times, biases can be faced by a multidisciplinary approach. In our case study, however, the building of the monuments and their quasi-immediate closure after the burial of the animal remains, strongly reduce the limits indicated before. The contexts here analysed were well preserved and did not show any evidence of heavy plundering: for these reason, we are inclined to use pollen and plant remains as proxy for the understanding of seasonality and rituals.

In this sense, we can consider the few plant remains as offerings in the burials here studied, in particular at structures 07/79 C1, 07/39 C3, and 07/110 C1. There is here a significant presence of fruits and other plant parts of a limited number of species, together with abundance of pollen of the daisy family. A special selection of fruits of *Rumex* is evident suggesting that they were expressively collected from the whole plants.

In the first two structures, anthers of Asteraceae (pollen clumps) were also found signalling the presence of flowers. Moreover, concentrations of macroremains and pollen are relatively high. This is true if we consider the open-air position of the stone monuments though it is not comparable to the very high levels common in rock shelters [35].

The prevalent taxa in the botanical records possess attractive features for humans. The flowers of the daisy family have scent, some of them have beautiful colours and many are used for their medicinal properties (for example, species of *Pulicaria* and *Artemisia*).

The fruits of *Rumex cyprius* Murb. and *Rumex vesicarius* L. give colour to the landscape. When their fruits are ripe, they become winged and purplish-red veined [81] (Fig. 11). Today, sorrel flowers in spring, from March to April, at any time after rain in the desert.

The whole plant is rich of constituents (flavonoids, C-glycosides, oxalic acid, tannins, mucilage, mineral salts and vitamin C). Particularly the leaves and seeds are collected and prepared fresh or as a powder for internal use, to treat liver diseases and as a laxative. Traditional medicine uses the plant as an antiscorbutic, appetiser, astringent, carminative, stomachic and tonic, and for jaundice. The leaves are eaten fresh and much appreciated for their acid taste [53].

## Methods

During the desktop phase, available and published data on stone monuments were entered in a GIS platform, in order to perform analysis on Landsat satellite imagery, together with high-resolution spots (Quickbird; Google Earth ©). In particular, we targeted the *corbeilles* (‘baskets’): circular platforms with slabs obliquely set around their external perimeter often with an associated standing stone. They appeared to be in spatial connection with rock art concentrations [44], with early excavations revealing their function as favoured *loci* for the deposition of cattle [33].

In the field, we focussed on a specific region, the northern Messak Settafet (Figs. 1 and 4). Survey was carried out on foot with sampling kept to the minimum. Areas of particular relevance were mapped by means of Differential GPS and ETS (Electronic Total Station) with the aim of creating 3D terrain models (DTM).

We excavated selected monuments trying to minimize our impact: when possible, we preserved the external perimeter of each structure, so as to simplify systematic post-excavation reconstruction. Archaeological materials (mostly pottery and lithics), animal bones and botanical remains were sampled for laboratory analysis, which included a systematic radiocarbon dating programme.

Full information about the methods adopted by the different disciplines involved is available in Text S1.

## Conclusions

Convincing evidence of a very early and enduring ritual in the central Sahara is provided by the Middle Pastoral monuments of the Messak with cattle remains and associated rock art. The exceptionality of our case study resides, we believe, in the multidimensional investigation of a phenomenon that we have known mainly for its extension in time and space e.g., [3], [26], [28], [33], [82], [83], with little understanding of its nature or complexity.

Our work shows how Middle Pastoral human groups settled along the lake shores of the Edeyen of Murzuq during the rainy season, moved with the arrival of the dry months towards the higher and water-richer areas of the Messak plateau. This seasonal transhumance allowed them to cope with strong variations in rainfall and environmental constraints. In the Messak, settlements were light and ephemeral–probably to favour rapid drifts to other areas as soon as water and pasture were exhausted. During these stays, Messak herders built stone monuments and performed specific, formalized, rituals centred primarily on bovines. The capillary construction of highly codified monumental structures over a large area indicates a ritual deeply rooted within these human groups and represents the material evidence of collective ceremonies. On the basis of monument density and rock art concentration (and in a few cases on the quantity and clustering of trapping stones), we identify within the Messak four main *loci* (Bedis, Tilizaghen, Taleschout, Tin Sharuma), apparently of greater importance, which could be considered places of memory [84], whose meaning was actualized and revitalized generation after generation [25].

Several stone structures yielded animal bones, mainly of domestic cattle (adults and males) at their highest meat yield. In some cases, especially in the Bedis and Tin Einessnis area, the concentration of hundreds of trapping stones together with the number of animals slaughtered suggest the gathering of many people. Once animals were killed and meat shared, the leftovers were burnt outside the structures and later placed in the monument. In several contexts, standing stones with engraved animals were erected, while scenes centred on bovines were carved on the wadi walls in the immediate vicinity.

On the basis of isotopic evidence, the buried animals showed to be ‘local’ to the Messak-Murzuq region, moving between geologically similar substrates (as reflected in the Sr isotope ratios) yet variable environments (in accordance with δ^13^C and δ^18^ O data), thus reinforcing the transhumance model on a seasonal basis. In some cases, our evidence shows how animals grazing on different pastural areas were buried in the same structure, so as to suggest the assembly of different groups to share the same monument.

The systematic presence of stone maces, inside or outside the structure, often next to the animal remains, is another highly standardized part of the scenario. Archaeobotanical data indicate–at least for the monuments analysed–the performing of the rituals at the very end of the dry season: sorrels and many daises bloom in winter and spring, and we may indicate April/May (for the overlap of the flowering of Asteraceae and the fructification of *Rumex*) as an approximate time frame. The total lack of *Rumex* pollen in the samples studied also indicates the end of its blooming season (late spring). Inside the monuments, the rarity of offered fruits may be related to their fragility, but their intrinsic characteristics–rarity, beauty, and medical properties–reflect the important value given to these plants.

Although it is impossible to archaeologically connect the rituals performed to specific ceremonial events (initiation, passage, wedding, transhumance, etc.), the gathering of different groups that involved feasting with the slaughtering of cattle might be considered a peculiar, distinctive trait of Middle Pastoral herders. It would be fascinating to place these events at the end of the dry period, just before the transhumance from the Messak plateau towards the Murzuq lowlands, when the rainy season allowed the dispersal of these groups over a large area.

Even if the emergence of ritual burials of domestic cattle has been seen as a social response to deteriorating environmental conditions and expression of collective identity [33] or, alternatively, as material manifestation of ‘rain-making’ ceremonies and indicator of increasing complexity within Neolithic herders [21], it is its persistence and codification across the centuries to characterize this ritual as a specific ideological trait of Saharan pastoralists, as shown by the Middle Pastoral groups of the Messak: a potential, evocative analogue for the “African Cattle Complex” as known today.

## Supporting Information

Text S1 **Background, methods and supplementary data of the different disciplines.**
(DOCX)

Text S2 **Survey in the Northern Messak Settafet.**
(DOCX)

Table S1 **Main features of the** ***corbeilles*** **, used for GIS analysis.**
(XLS)

Table S2 **Main features of the surveyed contexts in Northern Messak Settafet.**
(XLSX)

Table S3 **Database of the main rock art features, used for GIS analysis.**
(XLS)

Table S4 **Main features of quartzarenite stone maces.**
(DOC)

Table S5 Measurements of cattle and ovicaprines elements (in mm following von den Driesch 1976; * indicates approximate measurements).
(XLSX)

Table S6 **Skeletal element quantification in the main structures (NISP = Number of Identified Specimens, MNE = Minimum Number of Elements, MNI = Minimum Number of Individuals).** The elements indicating the presence of more than one individual are in bold and underlined.
(XLSX)

## References

[1] Marshall F, Hildebrand E (2002) Cattle before crops: The beginnings of food production in Africa. Journal of World Prehistory 16: 99–144.

[2] di Lernia S (in press) The emergence and spread of herding in Northern Africa: a critical reappraisal. In: Mitchell PJ, Lane PJ, editors. Oxford Handbook of African Archaeology. Oxford: Oxford University Press.

[3] Wendorf F, Schild R, editors (2001) Holocene settlement of the Egyptian Sahara. New York: Kluwer Academic/Plenum Publishers.

[4] Smith AB (2005) African Herders: Emergence of Pastoral Traditions. Walnut Creek: AltaMira Press.

[5] Gifford-Gonzalez D, Hanotte O (2011) Domesticating Animals in Africa: Implications of Genetic and Archaeological Findings. Journal of World Prehistory 24: 1–23.

[6] Hassan FA, editor (2002) Droughts, Food and Culture: Ecological Change and Food Security in Africa's Later Prehistory. New York: Kluwer/Plenum.

[7] Dunne J, Evershed R, Salque M, Cramp L, Bruni S, et al (2012) First Dairying in 'Green' Saharan Africa in the 5th Millennium BC. Nature 486: 390–394. 22722200 10.1038/nature11186

[8] Gifford-Gonzalez D (2000) Animal disease challenges to the emergence of pastoralism in sub-Saharan Africa. African Archaeological Review 17: 95–139.

[9] Clutton-Brock J (1989) The Walking Larder. Patterns of Domestication, Pastoralism, and Predation. London: Unwin Hyman.

[10] Herskovits MJ (1926) The cattle complex in East Africa. American Anthropologist 28: 230–272.

[11] Dupire M (1962) Peuls nomades: Etude Descriptive des Wodaabe du Sahel Nigérien. Paris: Institut d'Ethnologie.

[12] Evans-Pritchard EE (1940 ) The Nuer: a Description of the Modes of Livelihood and Political Institutions of a Nilotic Tribe. Oxford: Clarendon Press.

[13] Stenning DJ (1959) Savannah Nomads: a Study of the Wodaabe Pastoral Fulani of Western Bornu Province, Northern Region, Nigeria. London: Oxford University Press.

[14] Galaty JG (1989) Cattle and cognition: Aspects of Maasai practical reasoning. In: Clutton-Brock J, editor. The Walking Larder Patterns of Domestication, Pastoralism, and Predation. London: Unwin Hyman. pp 215–230.

[15] Lienhardt RG (1961) Divinity and experience. The religion of the Dinka. Oxford: Clarendon.

[16] Poland M, Hammond-Tooke D, Voigt L (2003) The abundant herds: a celebration of the Nguni cattle of the Zulu people. Cape Town: Fernwood.

[17] Blench R, MacDonald KC, editors (2000) The Origins and Development of African Livestock: Archaeology, Genetics, Linguistics, and Ethnography. London: UCL Press.

[18] di Lernia S, Tafuri MA (in press) Persistent deathplaces and mobile landmarks. The Holocene mortuary and isotopic record from Wadi Takarkori (SW Libya). Journal of Anthropological Archaeology.

[19] Kobusiewicz M, Kabacinski J, Schild R, Irish JD, Wendorf F (2009) Burial practices of the Final Neolithic pastoralists at Gebel Ramlah, Western Desert of Egypt. pp 147–174.

[20] Hildebrand E, Shea J, Grillo K (2011) Four middle Holocene pillar sites in West Turkana, Kenya. Journal of Field Archaeology 36: 181–200.

[21] Wendorf F, Schild R (1998) Nabta Playa and Its Role in Northeastern African Prehistory. Journal of Anthropological Archaeology 17: 97–123.

[22] di Lernia S, Zampetti D, editors (2008) La Memoria dell'Arte. Le Pitture Rupestri dell'Acacus tra Passato e Futuro. Firenze: All'Insegna del Giglio.

[23] Lhote H (1959) The Search for the Tassili Frescoes: The Story of the Prehistoric Rock-Paintings of the Sahara. New York: Dutton.

[24] Jelinek J (2003) Pastoralism, burials and social stratification in central Sahara. Les Cahiers de L'AARS 8: 41–44.

[25] di Lernia S, Gallinaro M (2010) The date and context of Neolithic rock art in the Sahara: engravings and ceremonial monuments from Messak Settafet (south-west Libya). Antiquity 84: 954–975.

[26] Applegate A, Gautier A, Duncan S (2001) The North tumuli of the Nabta Late Neolithic ceremonial complex. In: Wendorf F, Schild RA, editors. Holocene Settlement of the Egyptian Sahara, The Archaeology of Nabta Playa. New York: Kluwer Academic. pp. 468–488.

[27] Clark JD, Gifford-Gonzalez D, editors (2008) Adrar Bous: Archaeology of a Central Saharan Granitic Ring Complex in Niger. Tervuren: Royal Museum for Central Africa.

[28] Paris F (2000) African livestock remains from Saharan mortuary contexts. The Origins and Development of African Livestock: Archaeology, Genetics, Linguistics, and Ethnography 111–126.

[29] Roset JP (1987) NeÌolithisation, NeÌolithique et post-NeÌolithique au Niger nordoriental. International Journal of the French Quaternary Association 32: 203–214.

[30] Gifford-Gonzales D, Parham J (2008) The fauna from Adrar Bous and surrounding areas. In: Clark JD, Gifford-Gonzalez D, editors. Adrar Bous: Archaeology of a Central Saharan Granitic ring complex in Niger. Tervuren: Museée Royal de l’Afrique Centrale. pp. 313–353.

[31] Tauveron M, Ferhat N, Striedter KH (2009) Neolithic Domestication and Pastoralism in Central Sahara. The cattle necropolis of Mankhor (Tadrart Algérienne). In: Baumhauer R, Runge J, editors. Holocene Palaeoenvironmental History of the Central Sahara: CRC Press pp 179–186.

[32] Chaix L (2001) Animals as symbols. The bucrania of the grave KN24 (Kerma, Northern Sudan). In: Buitenhuis H, Prumme W, editors. Animals and Man in the Past. Groningen: ARC-Publicatie. pp 364–370.

[33] di Lernia S (2006) Building monuments, creating identity: Cattle cult as a social response to rapid environmental changes in the Holocene Sahara. Quaternary International 151: 50–62.

[34] Cremaschi M, Zerboni A (2011) Human communities in a drying landscape. Holocene climate change and cultural response in the central Sahara. In: Martini IP, Chesworth W, editors. Landscape and Societies. Dordrecht Heidelberg London New York: Springer Science. pp 67–89.

[35] Mercuri AM (2008) Human influence, plant landscape evolution and climate inferences from the archaeobotanical records of the Wadi Teshuinat area (Libyan Sahara). Journal of Arid Environments 72: 1950–1967.

[36] Mercuri AM (2008) Plant exploitation and ethnopalynological evidence from the Wadi Teshuinat area (Tadrart Acacus, Libyan Sahara). Journal of Archaeological Science 35: 1619–1642.

[37] Cremaschi M, di Lernia S (1999) Holocene climatic changes and cultural dynamics in the Libyan Sahara. African Archaeological Review 16: 211–238.

[38] di Lernia S (2002) Dry climatic events and cultural trajectories: adjusting Middle Holocene Pastoral economy of the Libyan Sahara. In: Hassan F, editor. Droughts, Food and Culture. New York: Kluver Academic/Plenum Publisher. pp 225–250.

[39] Tafuri MA, Bentley RA, Manzi G, di Lernia S (2006) Mobility and kinship in the prehistoric Sahara: Strontium isotope analysis of Holocene human skeletons from the Acacus Mts. (southwestern Libya). Journal of Anthropological Archaeology 25: 390–402.

[40] Biagetti S, di Lernia S (2003) Vers un modèle ethnographique-écologique d'une société pastorale préhistorique Saharienne. Sahara 14: 7–30.

[41] Anag G, di Lernia S (2007) The Archaeological Survey: Aims, Methodology and Results. In: Anag G, Cosentino L, Di Lernia S, editors. Edeyen of Murzuq Archaeological Survey in the Libyan Sahara. Firenze: All'Insegna del Giglio.

[42] Cremaschi M, di Lernia S (2000) Lasmo N-FC 174 Concession Area. The Messak Settafet Rescue Operation (Libyan Sahara): preliminary report. Department of Antiquities/Cirsa: Tripoli and Rome.

[43] Gallinaro M, Gauthier C, Gauthier G, Le Quellec J-L, Abdel Aziz S, et al (2012) The Messak Project. Cultural and Natural Preservation and Sustainable Tourism (south-western Libya). Antiquity Project Gallery

[44] Gauthier Y, Gauthier C (2004) Un exemple de relations monuments-art rupestre: "Corbeilles" et grands cercles de pierres du Messak (Libye). *Les Cahiers De L'AARS* 9: 45–63, pl. K–N.

[45] Ponti R (2001) Struttura megalitica nel Messak Settafet (Sahara libico). Sahara 13: 132–135.

[46] Ponti R (2003) Il tumulo di In-Habeter III (Sahara libico). Sahara 14: 161–166.

[47] Le Quellec J-L (1998) Art Rupestre et Préhistorie du Sahara. Le Messak Libyen. Paris: Bibliotheque Scientifique Payot.

[48] Lutz R, Lutz G (1995) The Secret of the Desert. The Rock Art of Messak Settafet and Messak Mellet, Libya (Das Geheimnis Der Wüste. Die Felskunst Des Messak Settafet Und Messak Mellet, Libyen). Innsbruck: Universtitätbuchhandlung Golf Verlag.

[49] Van Albada A, Van Albada AM (2000) La Montagne des Hommes-Chiens. Art Rupestre du Messak Libyen. Paris: Edition du Seuil.

[50] di Lernia S, Cremaschi M (1997) Processing quartzite in central Sahara: a case-study from In Habeter IIIA-Wadi Mathendusc (Messak Settafet, Libya). Man and Flint 225–232.

[51] Perego A, Zerboni A, Cremaschi M (2011) The geomorphological map of the Messak Settafet and Mellet (Central Sahara, SW Libya). Journal of Maps v2011: 464–475.

[52] Gasse F (2000) Hydrological changes in the African tropics since the Last Glacial Maximum. Quaternary Science Reviews 19: 189–211.

[53] Ozenda P (2000) Flore et végétation du Sahara. Paris: CNRS.

[54] White F (1983) The Vegetation of Africa. Paris: UNESCO.

[55] Zerboni A (2008) Holocene rock varnish on the Messak plateau (Libyan Sahara): chronology of weathering processes. Geomorphology 102: 640–651.

[56] Zerboni A, Trombino L, Cremaschi M (2011) Micromorphological approach to polycyclic pedogenesis on the Messak Settafet plateau (central Sahara): Formative processes and palaeoenvironmental significance. Geomorphology 125: 319–335.

[57] Cancellieri E, di Lernia S (in press) Middle Stone Age human occupation and dispersals. New data from the Messak plateau (SW Libya, central Sahara). Quaternary International.

[58] Anag G, Cremaschi M, Lernia SD, Liverani M (2002) Environment, Archaeology, and Oil: The Messak Settafet Rescue Operation (Libyan Sahara). African Archaeological Review 19: 67–73.

[59] Gallin A, Le Quellec JL (2008) Les ensembles céramiques du Bassin de Murzuq-une contribution de l'archéologie préventive à la connaissance du Messak. *Les Cahiers De L'AARS* 12: 71–88.

[60] Trevisan Grandi G, Mariotti Lippi M, Mercuri AM (1998) Pollen in dung layers from rockshelters and caves of Wadi Teshuinat (Libyan Sahara). Wadi Teshuinat Palaeoenvironment and Prehistory in South-Western Fezzan (Libyan Sahara) 7: 95–106.

[61] Graziosi P (1942) L'Arte Rupestre della Libia. Napoli: Edizioni della Mostra d’Oltremare.

[62] Jelinek J (2004) Sahara. Histoire de l’art rupestre libyen. Grenoble: Jérôme Millon.

[63] Corridi C (1998) Faunal remains from Holocene archaeological sites of the Tadrart Acacus and surroundings. Wadi Teshuinat Palaeoenvironment and Prehistory in South-western Fezzan (Libyan Sahara) 7: 89–94.

[64] Silvermann BW (1986) Density Estimation for Statistics and Data Analysis. Monographs on Statistics and Applied Probability. London: Chapman and Hall.

[65] Caneva I (1987) Pottery decoration in prehistoric Sahara and Upper Nile: A new perspective. BAR International Series 368: 231–254.

[66] Clark JD, Carter PL, Gifford-Gonzalez D, Smith AB (2008) The Adrar Bous Cow and African Cattle. In: Clark JD, Gifford-Gonzalez D, editors. Adrar Bous: Archaeology of a Central Saharan Granitic ring complex in Niger. Tervuren: Museée Royal de l’Afrique Centrale. pp. 355–368.

[67] Paris F (2000) African livestock remains from Saharan mortuary contexts. In: Blench R, MacDonald KC, editors. The Origins and Development of African Livestock: Archaeology, Genetics, Linguistics, and Ethnography. London: UCL Press. pp. 111–126.

[68] Bocherens H, Mashkour M, Billiou D, Pellé E, Mariotti A (2001) A new approach for studying prehistoric herd management in arid areas: intra-tooth isotopic analyses of archaeological caprine from Iran. Comptes rendus de l'Académie des Sciences Série 2: Sciences de la terre et des Planètes 332: 67–74.

[69] Smedley MP, Dawson TE, Comstock JP, Donovan LA, Sherril DE, et al (1991) Seasonal carbon isotope discrimination in a grassland community. Oecologia 85: 314–320. 28312034 10.1007/BF00320605

[70] Dansgaard W (1964) Stable isotopes in precipitation. Tellus 16: 436–468.

[71] Bentley RA (2006) Strontium Isotopes from the Earth to the Archaeological Skeleton: A Review. Journal of Archaeological Method and Theory 13: 135–187.

[72] Bekure S, De Leeuw PN, Grandin BE, Neate PJH (1991) Maasai herding: an analysis of the livestock production system of Maasai pastoralists in eastern Kajiado District, Kenya. ILCA Systems Study 4: 1–172.

[73] Cerling TE, Harris JM (1999) Carbon isotope fractionation between diet and bioapatite in ungulate mammals and implications for ecological and paleoecological studies. Oecologia 120: 347–363. 28308012 10.1007/s004420050868

[74] Balasse M, Ambrose SH (2005) Mobilité altitudinale des pasteurs néolithiques dans la vallée du Rift (Kenya): premiers indices de l’analyse du δ13C de l’émail dentaire du cheptel domestique. Anthropozoologica 40: 147–166.

[75] Florenzano A, Mercuri AM, Pederzoli A, Torri P, Bosi G, et al (2012) The significance of intestinal parasite remains in pollen samples from Mediaeval pits in the Piazza Garibaldi of Parma, Emilia Romagna, Northern Italy. Geoarchaeology 27: 34–47.

[76] Lézine AM (2007) Pollen records, postglacial. In: Elias SA, editor. Encyclopaedia of Quaternary Sciences: Elsevier. pp 2682–2698.

[77] Watrin J, Lézine AM, Hely C, Contributors a (2009) Plant migration and plant communities at the time of the 'green Sahara'. Comptes Rendu Geosciences 341: 656–670.

[78] Giraudi C, Mercuri AM, Esu D (2012) Holocene palaeoclimate in the northern Sahara margin (Jefara Plain, northwestern Libya). The Holocene

[79] Mercuri AM, Massamba N’siala I, Florenzano A (in press) Environmental and ethnobotanical data inferred from pollen of Gobero and the dried lakebeds in the surrounding area. In: Garcea E, editor. Gobero: the No-Return Frontier Archaeology and Landscape at the Saharo-Sahelian Borderland.

[80] Bunting MJ, Tipping R, Downes J (2001) “Anthropogenic” Pollen Assemblages from a Bronze Age Cemetery at Linga Fiold, West Mainland, Orkney. Journal of Archaeological Science 28

[81] Boulos L (2000) Flora of Egypt. Cairo, Egypt: Al Hadara Publishing.

[82] Ã–zbek M (2001) Cranial deformation in a subadult sample from DeÇ§irmentepe (Chalcolithic, Turkey). American Journal of Physical Anthropology 115: 238–244. 11424075 10.1002/ajpa.1078

[83] Wendorf F, Schild R, Applegate A, Gautier A (1997) Tumuli, cattle burials and society in the Eastern Sahara. Dynamics of Populations, Movements and Responses to Climatic Change in Africa 90–104.

[84] Lane PJ (in press) Archaeologies of East African pastoralist landscape: places and paths of memory. In: Broch-Due V, editor. Path Versus Place: Reconfiguring Nomads to Fit the State. Uppsala.

[85] Bronk Ramsey C (2009) Dealing with outliers and offsets in radiocarbon dating. Radiocarbon 51: 1023–1045.

