# Supplementary material for: Inside the “African Cattle Complex”: Animal Burials in the Holocene Central Sahara
Source: PLoS One. 2013 Feb 20;8(2):e56879. doi: 10.1371/journal.pone.0056879 (PMC3577651; doi:10.1371/journal.pone.0056879)
Supplement: Text S1 — Background, methods and supplementary data of the different disciplines. (DOCX) [file pone.0056879.s001.docx]

**SI TEXT 1: BACKGROUND, METHODS AND SUPPLEMENTARY DATA**

**1. GIS Analysis (Marina Gallinaro, Alessandro Perego, Andrea Zerboni and Savino di Lernia)**

***Geomorphology of the study area (Alessandro Perego, Andrea Zerboni)***

Due to the size of the study area, the geoarchaeological and geomorphological investigation of the Messak region was carried out on the basis of field surveys of the critical regions, integrated with the use of remote sensing data. In the region, thanks to the almost complete absence of vegetation, the Landsat and ASTER multispectral data provide good indications for distinguishing surface features and geomorphological units and a band ratio is a useful method for characterizing the properties of the land surface (e.g. Gad and Kusky 2006; Gani and Abdelsalam 2006; Perego et al. 2011). Landsat TM data acquired in 2000 and ETM+ data acquired in 2006 were analysed, both with 30m spatial resolution in multispectral bands. Mathematical ratios between bands revealed peculiar characteristics of the plateau, whereas small scale geomorphological features and wadi properties were checked on Google Earth and in many cases checked in the field. Morphological analyses were based on SRTM DEM (Shuttle Radar Topography Mission, Digital Elevation Model) data, acquired in 2000 with a spatial resolution of ca. 90 m. SRTM allowed the evaluation of terrain altitudes, delimiting valleys cut into the plateau, and extracting contour lines at 100 m vertical intervals. The following software was used: OSSIM Image Linker 1.8. (manipulation of satellite imagery); SAGA GIS 2.0.6 (digital elevation model data analysis); ArcGIS 9.2 (data rendering).

***Analysis of the* corbeilles *(Marina Gallinaro, Alessandro Perego, Andrea Zerboni and Savino di Lernia)***

Published information, fieldwork data and unpublished data referring to the *corbeille* monuments and rock art sites entered in two different databases (Tables S1 and S3), whose description is reported below.

The analyses were performed by means of ArcGIS 9 ver. 9.3 and included: descriptive statistics (Table S1 and S3), reported below together with the spatial analyses. These are the Average Nearest Neighbour (Clark and Evans 1954) discussed in the text and the Kernel Density (Silvermann 1986) displayed in figure 2 of the main text.

***Data of the* corbeille *monuments***

The database (Table S1) includes 197 records and is organized in six main blocks of data (A-F):

**A. Id and Locality** – general information of each *corbeille*:

1. ***Id structure***: univocal identifier number of the structure attributed by the latitudinal position from North to South;
2. ***Structure field code***: structure code identifying the structures surveyed or excavated during the Messak Ceremonial Monuments Project (MCMP), or during the rescue activities of the MSRS project;
3. ***Wadi name***: name of the closest wadi. Toponyms according to Van Albada and Van Albada 2000.

**B. Position and Topography** – general information about the geographical setting of the structure, referring to the geomorphological classification (cf. Geomorphology of the study area):

1. ***Topograpy****:*
   *Hamada*: location on the rocky surface of the plateau; the wadi bed is not easily accessible and requires specific tracks;
   *Wadi (in)*: location along the course of the wadi;
   *Wadi (out)*: location along the wadi, but outside the limit of its main course (e.g., on a gently rolling slope, on a gravel terrace…); the wadi bed is easily accessible.
2. ***Geomorphology***:
   *Outcrop*: sandstone outcrop on the plateau;
   *Desert pavement*: residual desert pavement on the plateau;
   *Endorheic depression*: small pseudo-karst depression on the top of the plateau;

*Slope:* gently rolling cliff;

*Fluvial scarp*: steep wadi bank;

*Wadi*: course of the wadi.

**C. Wadi Features** –information about the main characters of the closest portion of wadi:

1. ***Wadi Hierarchy****:*
   *Main*: main wadi;
   *Tributary*: secondary wadi, tributary of a main wadi;
   *Confluence*: confluence between two wadis;

*Mouth*: final part of a wadi, transition toward the pediment or the alluvial plain.

1. ***Wadi Geography****:
   Origin*: initial part of the wadi, corresponding to the confluence of ephemeral and minor first-order tributaries;
   *Middle*: middle course of the wadis; the valleys are deeply cut into the plateau and have a meandering course; steep valley flanks have an abrupt change in slope angle to a flat and wide wadi floor, which is covered by fluvial sediments (sand to gravel); gravel mega-bars are sometimes present;
   *End*: lower course of the wadi, at the transition to the pediment or the alluvial plain; the wadi is wide and flat, large meanders are covered by sand and gravel;
2. ***Wadi Morphology:*** the morphological setting of the wadi, within a radius of 1 km around the site:
   *Straight*: linear course;
   *Meandering*: meandering wadi; frequent changes in direction along a 2 km long transect, with a diameter of the wadi curve <1 km;
   *Wide curve*: wide bend of the wadi, diameter of the wadi curve >1 km.

**D. Monument features** – information about the architectural features of the monuments:

1. ***Type*:** simplified typology of the *corbeille* monuments; the structures are characterised by a high level of variability, dependent on the available raw material, their location in the landscape and the surface morphology. Three main types were identified:

*Simple*: structure with a circular perimeter made of slabs obliquely set in the ground, often with a central standing stone;

*Double ring*: structure with an external perimeter and an inner ring, with or without standing stone;

*Complex*: structure with adjoining external annexes or standing stones.

1. ***Size:*** diameter (corbeilles have a circular shape):

*Small***:** <3m;

*Medium***:** 3-5m;

*Large***:** >5m.

1. ***Elevation***: structure height, above ground:

*Flat*: <30cm;

*Slightly elevated*: 30-50cm;

*Elevated*: >50cm.

1. ***Stelae****:* presence/absence and characteristics of the standing stones:

*Absent*;

*Single*: only one standing stone;

*Single engraved*: one standing stone with engravings;

*Multiple*: ≥ 2 standing stones;

*Multiple engraved*: ≥ 2 standing stones, at least one of which, engraved.

**E. Intersite feature** – aspects of the relationships between structures and/or geomorphological features, as derived from descriptive statistics and remote sensing:

1. ***Reciprocal minimum distance***: assessment of the reciprocal visibility and possible correlation between the structures, calculated on the basis of the distance between each *corbeille* and the nearest one; five main classes have been statistically identified:

*Aggregated*: minimum distance <30m;

*Thinned:* minimum distance 30-90m;

*Isolated*: minimum distance 90-900m;

*Remote*: minimum distance 900-2500m;

*Very remote*: minimum distance >2500m.

1. ***Distance from wadi bank***: evaluation on the basis of remote sensing and field control. The linear distance from the wadi wall is estimated for each *corbeille*; the resulting statistical groups define four main classes:

*Very close:*  On the wadi edge: <30m;

*Close*: Adjacent to the wadi bank: 31-75m;

*Far*: In proximity of the wadi bank: 76-175m;

*Very far.* Far from the wadi bank*:* >175m.

1. ***Accessibility*** to the *corbeille* location: it is defined on the basis of remote sensing analysis and fieldwork control, by considering the geomorphological features within a radius of 2.5 km around the structure; three classes are defined:

*Hard*: *corbeille* with a surrounding ground steep for more than 70%;

*Medium*: *corbeille* with a surrounding ground steep <70%; surrounding ground plain/low degree slope <90%;

*Easy*: *corbeille* with a surrounding ground plain/low degree slope >90%.

**F. Data Source –** general information about the source of data and their reference.

***Analysis of rock art evidences (Marina Gallinaro)***

The rock art of the Messak region is extremely rich, particularly for the Pastoral period (Le Quellec 1998, Jelinek 2004, Van Albada and Van Albada 2000). As a general catalogue has not been established, so far, the generation of the database required managing a huge and heterogeneous quantity of information. Data have been extracted from: i) published information, sometimes lacking a clear spatial location of sites (Graziosi 2005, Jacquet 1988, Jelinek 1984, 1985a, 1985b, Le Quellec 1998, Lutz 1995, Pesce 1967, Van Albada and Van Albada 1990, 1992, 1993, 1994, 2000); ii) an unpublished set of spatially located data kindly provided to us by Axel and Anne-Michelle Van Albada; iii) data recorded during the MCMP fieldwork in 2007-2010.

In order to assess the distribution of scenes and subjects referable to a fully Pastoral period and their correlation with the *corbeilles*, the analysis focused on 102 scenes or palimpsests of scenes clearly referable, according to di Lernia and Gallinaro (2010), to a Middle Pastoral phase, for subject matter and style.

The following information was inserted for each scene in Table S3:

1. ***Id structure***: univocal identifier number of the scene attributed by the latitudinal position from North to South;
2. ***Wadi name***: name of the wadi where the engravings occur. Toponyms according to Van Albada and Van Albada 2000;
3. ***Site***: sites are named according to the reference: in Van Albada and Van Albada 2000 (wadi + km from wadi origin, e.g.: Isser km 42), or the site code in Jelinek 1985a, 1985b (Wadi shortening + site nr., e.g.: TW1), or the site code of the MCMP (year/nr. e.g.: 08/290); some “historically known” sites have a proper name (this is the case of: Garamantian Apollo; Mathendous and other sites quoted in Graziosi 2005, e.g. In Habeter II).
4. ***Scene code***: this code refers to the rock art iconography, following a classification aimed at highlighting the connections with generic life or potentially cultual scenes. Seven classes have been defined:

*1. Bovids* – cattle or cattle herds;

*2. Pastoral life* – association between humans and cattle;

*3. Transhumance* – herders and cattle on the move, with loads and/or ornaments;

*4. Simple mask scenes* – bovid-masked herders together with or without one or more cattle;

*5. Complex ritual scenes* – potentially ritual scenes, featuring a complexity of actions (e.g. so-called “dance scenes” or “processions”);

*6. Bovid sacrifice* – potentially ritual scenes, where a capsized bovid is surrounded by masked humans;

*7. Stereotipal ritual scene* – potentially ritual scenes, where richly attired women, tethering decorated bovids, surround a male represented in smaller size (sometimes interpreted as “marriages”).

***References***

Clark, PJ, Evans FC (1954) Distance to Nearest Neighbor as a Measure of Spatial Relationships in Populations. Ecology 35: 445–453

di Lernia S, Gallinaro M (2010) Dating Saharan rock art. Pastoral Neolithic ceremonial sites and associated engravings from Messak Settafet (SW Libya). Antiquity 84 (326): 954–975.

Gad S, Kusky T (2006) Lithological mapping in the Eastern Desert of Egypt, the Barramiya area, using Landsat Thematic Mapper (TM). Journal of African Earth Science 44: 196–202.

Gani NDS, Abdelsalam MG (2006) Remote sensing analysis of the Gorge of the Nile, Ethiopia with emphasis on Dejen-Gohatsion region. Journal of African Earth Science 44: 135–150.

Graziosi P (2005) Arte rupestre del Fezzan. Missioni Graziosi 1967 e 1968. Firenze: Ist. Italiano di Preistoria.

Le Quellec JL (1998) Art rupestre et prehistoire du Sahara. Le Messak Libyen. Paris: Editions Payot et Rivages, Bibliothèque Scientifique Payot.

Lutz R, Lutz G (1995). The Secret of the Desert. The Rock Art of the Messak Settafet and Messak Mellet, Libya. Innsbruck: Golf Verlag.

Jacquet G (1978) Au cœur du Sahara libyen, d'étranges gravures rupestre. Archéologia 123: 40-51

Jelínek J (1984) Mathrndush, In Galghien. Two important Fezzanese Rock Art sites. Part I. Anthropologie XXII(2): 117-170.

Jelínek J (1985a) Tilizahren, the key site of the Fezzanese Rock Art. Part I. Anthropologie, XXIII (2): 125-165.

Jelínek J (1985b) Tilizahren, the key site of the Fezzanese Rock Art. Part II. Anthropologie, XXIII (3): 223-275.

Jelínek J (2004) Sahara. Histoire de l’art rupestre libyen. Editions Jérôme Millon.

Perego A, Zerboni A, Cremaschi M (2011) Geomorphological Map of the Messak Settafet and Mellet(Central Sahara, SW Libya). Journal of Maps: 464-475

Pesce A (1967) Segnalazione di nuove stazioni d'arte rupestre negli Uidian Telìssaghen e Matrhandùsc (Messak Settafet, Fezzan). Rivista di Scienze Preistoriche 22 (2): 393-416

Silvermann BW (1986) Density Estimation for Statistics and Data Analysis. Monographs on Statistics and Applied Probability. London: Chapman and Hall,

Van Albada A, Van Albada AM (1990) Gravures du Messak Settafet. Sahara 3: 89-94

Van Albada A, Van Albada AM (1992) Les gravures rupestres neolithiques du sahara central. Archeologia 275: 22-33

Van Albada A, Van Albada AM (1993) Hommes, animaux et legendes de la prehistoire Fezzanaise. Archeologia 290: 40-49

Van Albada A, Van Albada AM (1994) De nombreux centres culturels. Les dossier d'Archeologie 197: 22-33

Van Albada A, Van Albada AM (2000) La Montagne des Hommes-Chiens. Art rupestre du Messak Libyen. Paris: Edition du Seuil.

-----------------------------------------------------------------------------------------------------------------

**2. Faunal remains (Francesca Alhaique)**

A total of 25,345 animal remains were collected from 30 features belonging to 22 monuments excavated in the Messak region between 2000 and 2009. The assemblage is in general very fragmented and poorly preserved and only 9.5% of the specimens could be identified to species level or assigned to higher taxonomic or size categories (Tab. 4 main text).

For the zooarchaeological analysis the different features have been considered separately in order to evidence possible differences within the structures (e.g., differential distribution of species, body parts, frequency and intensity of burning).

Species have been identified on the basis of the reference collections of the Zooarchaeology Laboratories of the Sapienza Università di Roma and of the Università degli Studi della Tuscia (Viterbo), of the Istituto Italiano di Paleontologia Umana (Rome), and of the Museo di Storia Naturale, Sezione di Zoologia “La Specola” of the Università di Firenze (Florence) as well as on published sources (e.g., Barone 1981, 1995, Gabler 1985, Peters 1986).

When fragmentation did not allow specific attribution of the remains, the specimens were classified according to size categories. For this study “Large ungulate” included domestic and wild cattle, equids, as well as other possible wild large bovids; ovicaprines, small gazelles, and suids were considered “Small ungulate”. However, on the basis the faunal composition of the assemblage analyzed, it is very likely that most, if not all, the large ungulate specimens belong to *Bos taurus* (except in 07/39 C1 where they may probably be referred to *Equus* sp.) and the small ungulates to *Ovis vel Capra*. “Small mammal” comprised animals of the size of lagomorphs, small felids and canids; “Microfauna” included small rodents and reptiles.

In the quantification of the assemblages both identifiable and unidentifiable fragments where counted in order to assess fragmentation in the different samples. Minimum Number of Elements (MNE) was calculated on the basis of the most common portion in each skeletal element also taking into account age and size of the specimen. Minimum Number of Individuals (MNI) was calculated on the basis of the most common element again taking into account age and size of the individuals. MNE and MNI, when different features belonged to the same monument from an archaeological point of view, were calculated considering the assemblage both as a single unit and as separate entities in order to check if possibly parts of the same animal had been deposited in the different sectors of the structure. Considering the species identified, when analyzing body part frequencies, *Bos taurus* have been grouped together with large ungulate (Equid and large ungulate in 07/39 C1); the same occurred for *Ovis vel Capra* and small ungulates.

Because of the fragmentary state of the samples, measurements could be taken only of a few complete portions following von den Driesch (1976) (SI Tab. 5).

Aging of cattle and ovicaprines was based on Barone (1981, 1995) and Payne (1973). In the case of *Bos taurus* such determination was revealed to be problematic because of a discrepancy between the information provided by tooth eruption and, especially, wear and long bone epiphyseal fusion. However, it was decided to consider this bias as produced by natural causes (e.g, more rapid tooth wear produced by grazing on a coarse substrate possibly associated to slower maturation rate of the skeleton) rather than the result of the contemporaneous sacrifice of two animals, one providing *only* the head, the other *only* skeleton, also because in almost all the structures both portions indicate the presence of a single individual. Furthermore, this explanation seems to be supported by the fact that such discrepancy increased with age.

Each single specimen, identifiable or not, was inspected for human, animal or other natural modifications. Intensity of burning was quantified on the basis of a simplified version of Stiner’s (Stiner et al 1995) codes (0=not burned, 1=slightly burned <half carbonized, 2= lightly burned >half carbonized, 3= fully carbonized, 4=partially or totally calcined).

***References***

Barone R (1995) Anatomia comparata dei mammiferi domestici. Volume 1 Osteologia. Bologna: Edagricole.

Barone R (1981) Anatomia comparata dei mammiferi domestici. Volume 3 Splancnologia. Bologna: Edagricole.

Gabler K-O (1985) Osteologische Unterscheidungsmerkmale am postkranialen Skelett zwischen

Mähnenspringer (*Ammotragus lervia*), Hausschaf (*Ovis aries*) und Hausziege (*Capra hircus*). Diss. med. vet., LMU-München.

Payne S (1973) “Kill-off patterns in sheep and goats: the mandibles from Aşvan Kale”. Anatolian Studies 23: 281-303.

Peters J (1986) Osteomorphology of the appendicular skeleton of African buffalo, *Syncerus caffer* (Sparrman, 1779) and Cattle, *Bos primigenius* f. *taurus* (Bojanus, 1827). Occasional Papers, Laboratorium voor Paleontologie. Rijksuniveristeit Gent, No.1.

Stiner MC, Weiner S, Bar-Yosef O, Kuhn SL (1995) Differential burning, fragmentation, and preservation of archaeological bone. Journal of Archaeological Science 22: 223-237.

Von den Driesch A (1976) A guide to the measurement of animal bones from archaeological sites (Peabody Museum Bulletin 1), Cambridge.

-----------------------------------------------------------------------------------------------------------------

**3. Isotope study (Mary Anne Tafuri, Marie Balasse, Paul Fullagar)**

*Carbon isotope ratio (Marie Balasse)*

The relative proportion of C_3_ and C_4_ plants has not been specifically established for the Libyan desert. On a wider scale, the Sahara desert vegetation is dominated by C_4_ plants, which are favoured in habitats characterized by elevated temperature during the growing season, aridity and/or high direct irradiance (Ehleringer et al., 1997). However, a more humid climate in the past may have created conditions for a better representation of C_3_ plants (Cremaschi et al., 2010). C_3_ and C_4_ plants are characterized by different carbon isotope ratios. Modern C_3_ plants have δ^13^C values averaging -27 ‰ and ranging from -37 ‰ in forest understories to -20 ‰ in hot and sunny habitats (Kohn, 2010). The δ^13^C values of modern C_4_ plants are about -12.5 ‰ in average and are comprised between -16 ‰ and -9 ‰ (Vogel et al., 1978; Cerling et al., 2003). In ancient ecosystems, the δ^13^C values of plants may be considered approximately 1.5 ‰ higher, due to the fossil fuel effect observed since industrial times in the δ^13^C of atmospheric CO_2_ and inherited in the δ^13^C of terrestrial plants (Francey et al., 1999; Cerling & Harris, 1999). The δ^13^C value of tooth enamel bioapatite carbonate is inherited from the whole diet (Ambrose & Norr, 1993). In large ruminants mammals, a 14.1‰ isotope enrichment (ε*) of ^13^C was measured between diet and enamel bioapatite (Cerling & Harris, 1999). Therefore the measurement of animal skeleton δ^13^C provides an indirect estimation of the proportion of C_3_ and C_4_ plants in the habitat, although this information is filtered through the species diet preferences. Given the strong relation between type of plants and environment, it might also provide palaeoenvironmental proxies.

Relative proportion of C_3_ and C_4_ plants to the diet can be calculated using a 14.1 ‰ isotope enrichment (ε*) of ^13^C between diet and enamel bioapatite (Cerling & Harris, 1999). This calculation lacks precision for several reasons: first, the mean worldwide value for C_3_ plants (-27‰) may not be a good reference for desert plants usually characterized by slightly higher δ^13^C values (Kohn, 2010) and consequently our calculated relative proportion of C_4_ plants may be slightly overestimated; second, the highest and lowest values of these cyclical sequences are attenuated by the delay of enamel mineralization (Balasse, 2002; Passey & Cerling, 2002; Zazzo et al., 2005); third, different digestibility of C_3_ and C_4_ plants might lead to slightly different fractionation factors and consequently different representation in the final δ^13^C values in mixed-feeding herbivores (Codron et al., 2011); fourth, when interpreting the data, one must keep in mind that cattle may be very selective feedersl and consequently the proportion of C_3_ and C_4_ plants in their diet may not always accurately reflect their relative proportion in the pasture area (Balasse & Ambrose, 2005).

*Oxygen isotope ratio (Marie Balasse)*

The oxygen isotope composition (δ^18^O) of enamel bioapatite phosphate and carbonate precipitates in isotopic equilibrium with body water. Body water tracks the oxygen isotopic composition of ingested water, including drinking water and water from plants, therefore indirectly meteoric water (Longinelli, 1984; Luz et al., 1984; Flanagan and Ehleringer, 1991). Seasonal variations in the δ^18^O of precipitation (Rozanski et al., 1993) are recorded in enamel during tooth mineralization and can be reconstructed through sequential analysis along tooth growth axis (Fricke & O’Neil, 1996; Balasse et al., 2003). Concomitant analysis of intra-tooth variations in enamel bioapatite δ^13^C and δ^18^O values permits the investigation of the composition of diet at a seasonal scale (Balasse et al., 2012).

*Strontium isotope ratio (Mary Anne Tafuri)*

The strontium isotopic ratio (^87^Sr/^86^Sr) in the inorganic fraction of archaeological skeletons is used to assess past mobility and residential patterns (Ericson 1985, Koch et al., 1994). The bioavailable strontium absorbed in the tissues reflects that of the plant (or meat) eaten, which in turn replicates that of soil and water as derived by the geological background of the lived-in environment. Some of the ^87^Sr content of bedrock is formed by decay of rubidium (^87^Rb), since formation of the rock, hence old rocks will generally have higher ^87^Sr than younger ones (for a discussion see Bentley 2006). Previous Sr isotope analysis of archaeological animals were performed by Hoppe et al. (1999) to assess mammoth migration, while Balasse et al (2002) and Bentley and Knipper (2005) used sequential C and O analysis and associated ^87^Sr/^86^Sr to explore seasonal mobility and transhumance respectively in Pastoral South African herders and Neolithic LBK groups.

Strontium isotope analysis (87Sr/86Sr) of human and animal remains from Late Acacus and Pastoral sites in SW Libya (Tafuri et al., 2006, di Lernia and Tafuri in press) has been used to reconstruct human mobility and social practices, and has proven to successfully discriminate within the geological complexity of the Libyan Sahara.

*Methods of sampling, pre-treatment and analysis (Mary Anne Tafuri, Marie Balasse, Paul Fullagar)*

For stable C and O isotope analysis we collected tooth enamel through 1-mm-wide grooves that run perpendicular to the tooth growth axis. Tooth surfaces were cleaned by abrasion with a tungsten drill bit. Enamel was sampled sequentially with a diamond drill bit. Each sample is a 1-mm-wide groove perpendicular to the tooth growth axis, taken through the whole thickness of the enamel layer. Enamel powder was pre-treated for isotopic analysis as described in Balasse et al. (2003). Stable isotope analyses were performed at the SSMIM at the MNHN, Paris, with technical support by Joël Ughetto-Monfrin (UMR 7209 CNRS). Enamel samples weighing approximately 600 μg were reacted with 100% phosphoric acid at 70°C for 240 seconds in individual vessels in an automated cryogenic distillation system (Kiel IV device), interfaced with a DeltaVAdvantage isotope ratio mass spectrometer. Over the period of analysis of these enamel samples, 48 analyses of our laboratory internal carbonate standard (Marbre LM) gave a mean δ^13^C value of 2.03 ± 0.02 ‰ (1σ) (theoretical value normalized to NBS 19 = 2.13 ‰) and a mean δ^18^O value of -1.55 ± 0.09 ‰ (1σ) (theoretical value normalized to NBS 19 = -1.83 ‰). The analytical precision within each run, calculated from 8 measurements of the standard Marbre LM, varies from 0.01 to 0.02 ‰ for δ^13^C and from 0.02 to 0.14 for δ^18^O.

For Sr isotope ratio, analyses were carried out at the Department of Geological Sciences at the University of North Carolina, Chapel Hill. Strontium was separated from dissolved samples by ion exchange chromatography using Eichrom Sr-Spec resin. Strontium was analyzed in dynamic multi-collector mode with a VG Sector 54 thermal ionization mass spectrometer. Internal precision, percent standard error for ^87^Sr/^86^Sr, is typically 0.0006 to 0.0009%, hence data are reported to 4 decimal places. Sr carbonate standard SRM 987 was analyzed 62 times during the year of analysis: mean ^87^Sr/^86^Sr ratio = 0.710270 ± 0.000018 (2 standard deviations). All ^87^Sr/^86^Sr ratios from the UNC-CH laboratory are reported relative to a value of 0.710250 for SRM 987.

***References***

Ambrose SH, Norr L (1993) Experimental evidence for the relationship of the carbon isotope ratios of whole diet and dietary protein to those of bone collagen and carbonate. In: J.B. Lambert & G. Grupe, eds. *Prehistoric Human Bone. Archaeology at theMolecular Level*. Berlin: Springer-Verlag, pp. 1-37.

Balasse M (2002) Reconstructing dietary and environmental history from enamel isotopic analysis: time resolution of intra-tooth sequential sampling. International Journal of Osteoarchaeology 12: 155-165.

Balasse M (2003) Potential biases in sampling design and interpretation of intra-tooth isotope analysis. International Journal of Osteoarchaeology 13: 3-10.

Balasse M, Boury L, Ughetto-Monfrin J, Tresset A (2012) Stable isotope insights (δ^18^O, δ^13^C) into cattle and sheep husbandry at Bercy (Paris, France, IV^th^ millennium BC): birth seasonality and winter leaf foddering. Environmental Archaeology 17: 29-44.

Balasse M, Smith AB, Ambrose SH, Leigh SR (2003). Determining sheep birth seasonality by analysis of tooth enamel oxygen isotope ratios: the Late Stone Age site of Kasteelberg (South Africa). Journal of Archaeological Science 3: 205-215.

Bentley RA (2006) Strontium Isotopes from the Earth to the Archaeological Skeleton: A Review. Journal of Archaeological Method and Theory 13 (3): 135-187.

Bentley RA, Knipper C (2005) Transhumance at the early Neolithic settlement at Vaihingen (Germany). Antiquity 79 (306) Project Gallery.

Cerling TE, Harris JM (1999) Carbon isotope fractionation between diet and bioapatite in ungulate mammals and implications for ecological and paleoecological studies. Oecologia 120: 347-363.

Cerling TE, Harris JM, Passey B (2003) Diets of East African bovidae based on stable isotope analysis. Journal of Mammalogy 84 (2): 456-470.

Codron D, Codron J, Sponheimer M, Bernasconi SM, Clauss M (2011) When animals are not quite what they eat: diet digestibility influences ^13^C-incorporation rates and apparent discrimination in a mixed-feeding herbivore. Canadian Journal of Zoology 89: 453-465.

Cremaschi M, Zerboni A, Spötl C, Felletti F (2010) The calcareous tufa in the Tadrart Acacus Mt. (SW Fezzan, Libya). An early Holocene palaeoclimate archive in the central Sahara. Palaeogeography, Palaeoclimatology, Palaeoecology 287: 81**-**94.

di Lernia S, Tafuri MA (2012). Persistent deathplaces and mobile landmarks. The Holocene mortuary and isotopic record from Wadi Takarkori (SW Libya). Journal of Anthropological Archaeology: in press

Ehleringer JR, Cerling TE, Helliker BR (1997) C_4_ photosynthesis, atmospheric CO_2_, and climate. Oecologia 112: 285-299.

Flanagan LB, Ehleringer JR (1991) Stable isotope composition of stem and leaf water: application to the study of plant water use*.* Functional Ecology 5: 270-277.

Francey RJ, Allison CE, Etheridge DM, Trudinger CM, Enting IG, Leuenberger M, Langenfelds RL, Michel E, Steele LP (1999) A 1000-year high precision record of δ^13^C in atmospheric CO2. Tellus 51 B: 170-193.

Fricke HC, O’Neil JR (1996) Inter- and intra-tooth variation in the oxygen isotope composition of mammalian tooth enamel: some implications for palaeoclimatological and palaeobiological research. Palaeogeography, Palaeoclimatology, Palaeoecology 126: 91-100.

Hoppe KA, Koch PL, Carlson RW, Webb DS (1999) Tracking mammoths and mastodons: Reconstruction of migratory behavior using strontium isotope ratios. Geology 27: 439-442.

Kohn MJ (2010) Carbon isotope compositions of terrestrial C_3_ plants as indicators of (paleo)ecology and (paleo)climate. Proceedings of the National Academy of Sciences of the United States of America 107: 19691-19695.

Longinelli A (1984) Oxygen isotopes in mammal bone phosphate: a new tool for paleohydrological and paleoclimatological research. Geochimica et Cosmochimica Acta 48: 385-390.

Luz B, Kolodny Y, Horowitz M (1984) Fractionation of oxygen isotopes between mammalian bone-phosphate and environmental drinking water. Geochimica et Cosmochimica Acta 48: 1689-1693.

Passey BH, Cerling TE (2002) Tooth enamel mineralization in ungulates: implications for recovering a primary isotopic time-series. Geochimica et Cosmochimica Acta 66: 3225-3234.

Rozanski K, Araguas-Araguas L, Gonfiantini R (1993) Isotopic patterns in modern global precipitation. In: Stwart PK, Lohmann KC, McKenzie J, Savin editors. Climate change in continental isotope records. Washington: American Geophysical Union. pp. 1-36.

Tafuri MA, Bentley AR, Manzi G, di Lernia S (2006) Mobility and kinship in the prehistoric Sahara: strontium isotope analysis of Holocene human skeletons from the Acacus Mts. (southwestern Libya). Journal of Anthropological Archaeology 25: 390-402.

Vogel JC, Fuls A, Ellis RP (1978) The geographical distribution of Kranz grasses in South Africa. South African Journal of Science 74: 209-215.

Zazzo A, Balasse M, Patterson WP (2005) High-resolution δ^13^C intra-tooth profiles in bovine enamel: Implications for mineralization pattern and isotopic attenuation? Geochimica et Cosmochimica Acta 69: 3631-3642.

-----------------------------------------------------------------------------------------------------------------

**4. Archaeobotanical analyses (Anna Maria Mercuri)**

Plant macroremains were collected at naked eye in the field, except for sample mc5 that was dry-sieved through a series of 10, 0.5 and 0.2 mm sieves. Macroremains were gently sieved with a strainer to eliminate fine sand and then sorted with a brush under a Wild M10 stereomicroscope at 20x magnifications. Identifications were made at 50x magnifications.

About 5 grams of sediment were treated for pollen extraction by using tetra-Na-pyrophosphate, HCl 10%, sieving with a nylon 7 μm mesh, acetolysis, heavy liquid separation (Na-metatungstate hydrate), HF 40% and ethanol. *Lycopodium* tablets were added for calculation of concentrations, expressed as pollen per gram (p/g). Residues in glycerol were mounted in permanent slides (Florenzano et al. 2012). A mean of 250 pollen grains per sample were counted at 400x and 1000x magnifications. Percentages are calculated on a pollen sum which includes all pollen grains. Pollen nomenclature follows the African Pollen Database, and the interpretative approaches in Giraudi et al. (2012). Ecology and phytogeographical affinity of plants are according to Boulos (2000), Ozenda (2000) and Watrin et al. (2009).

Pollen was identified at 400x and 1000x magnifications, with the help of the reference collection and atlases.

***

**Supplementary data**

Palynofacies of samples from structures 07/39 C3 and 07/79 C1 are characterised by small charcoal particles and mineral inclusions. Samples p1 and p3 from structure 07/39 C3 also contain some large charcoal particles of 100-150 μm that may be related to near-site (local?) burning activity (Carcaillet 2007). In these two structures, pollen concentrations are good ranging from ca. 1000 p/g (in p4) to ca. 3500 p/g (in p5). Contrarily, the palynofacies of samples from structure 07/110 C1 are very poor and pollen concentration is low (ca. 800 p/g). This reveals the different content of organic matter that is lower in 07/110 C1 than in the other structures.

Pollen preservation was adequate to obtain reliable results. However, they were commonly affected by the deterioration proper of sandy archaeological deposits as a result of post-depositional disturbance including compaction of sediments (broken or folded grains) or hydratation-dehydratation cycles (thin-white grains, especially in p1 of structure 07/39 C3). Clumps of single pollen type were observed in sample p2, and less in p1, and are indication of the presence locally of flowers or excrements (Mercuri 2008).

Among the NPPs (Non Pollen Palynomorphs), there are one Paniceae phytolith (in p3), few algal elements (in p1, and p5) and clamydospores of *Glomus*. The latter one was especially found in p4, while it was in traces in p1 and in p6-p7 (07/110 C1). *Glomus* is a fungus living within roots of plants (endomychorizza) and has typical smooth and spherical spores. Its increase in sediments may be related to erosion processes.

***References***

Carcaillet C (2007) Charred particle analysis. In: Elias SA editor. Encyclopedia of Quaternary Science, vol. 2. Amsterdam: Elsevier. pp. 1582–1593.

Mercuri AM (2008) Plant exploitation and ethnopalynological evidence from the Wadi Teshuinat area (Tadrart Acacus, Libyan Sahara). Journal of Archaeological Science 35(6): 1619–1642.

-----------------------------------------------------------------------------------------------------------------
