# Supplementary material for: Inside the “African Cattle Complex”: Animal Burials in the Holocene Central Sahara
Source: PLoS One. 2013 Feb 20;8(2):e56879. doi: 10.1371/journal.pone.0056879 (PMC3577651; doi:10.1371/journal.pone.0056879)
Supplement: Text S2 — Survey in the Northern Messak Settafet. (DOCX) [file pone.0056879.s002.docx]

SI TEXT 2: SURVEY IN THE NORTHERN MESSAK SETTAFET

It should be underlined as remote sensing analysis – also on high resolution spots – can only help to identify stone structures, whose specific typology must be assessed in the field. This is even more evident for the Messak, whose desert pavement made of barren rocks makes any chromatic or shape recognition difficult.

When in the field, the distribution of structures made difficult, without excavation, the very definition of ‘site’. In principle, very close structures were labelled under the same code number and then progressively numbered (e.g., 07/39 C1…; codes for the principal types of structures are: C Corbeille; T Tumulus; SS Stone Structure; SR Stone Ring; SP Stone Platform): however, it appears clear, after excavations, that this criterion was not always valid, as also indicated by previous research on stone funerary monuments [[1](#_ENREF_1)]. In other cases, intensive areas of fieldwork were labelled and all the structures were given a progressive number (e.g., Tin Einessnis area 1, site 00/556). These differences refer to two distinct phases of fieldwork, carried out respectively on 2000 and 2007-2009: i) the former, a rescue operation (Messak Settafet Rescue Survey, MSRS: [[2](#_ENREF_2)]) consisted of a linear, 70 km-long, survey along a road to be built, with intensive survey in the immediate vicinity (Tin Einessnis 1 & 2); ii) the latter was conceived to target *corbeilles* and associated monuments and covered 6 areas. This fieldwork added to the isolated excavation done in the 1990s at In Habeter IIIa [[3-5](#_ENREF_3)]. The results of earlier research are presented first.

*In Habeter IIIa*

The area of In Habeter, southern end of the massif close to the famous Mathendous rock art site, was investigated in the 1990s [[3-6](#_ENREF_3)]. In particular, we excavated the foundation pit of a rough standing stone (ca. 70cm in height, 40 cm wide), containing two pots and the poorly preserved remains of a single, adult *Bos taurus* male, dated to 5213 ± 80 yr BP (GX 19108-AMS). This context is not directly related to *corbeille*-like structures, but given its content it will be included in the general discussion.

*Tin Einessnis 1*

The area (650x300 m) extends along the western bank of Wadi Tin Einessnis, steeper and higher in this point. It was selected for the concentration of stone structures, engravings and trapping stones. These stones, often represented on the wadi walls, feature one or two notches, less frequently a continuous groove, and are used either to trap wild faunas or tether domestic animals [[7](#_ENREF_7)]. The DGPS mapping recorded 6 stone structures, mainly tumuli and four settlement areas all of Final Pastoral/Early Garamantian age. The persistence of human presence over millennia supports the idea of a favourable area from an environmental point of view also thanks to the presence of several gueltas. The use and re-use of structures and monuments makes this area an important place of memory. We excavated structure 00/300 (looted), whereas structures 00/301 and 00/301a were apparently well preserved.

*Tin Einessnis 2*

The area shows among the richest concentration of rock art in the entire Messak [[8](#_ENREF_8)]. The mapped area is approximately 400x300 m. It includes the wadi bank, a small valley delimited by two minor tributaries, and two large quartzite outcrops. Several stone structures were mapped (flat and conical tumuli, stone rings, stone accumulations). Part of these structures should be related to both living and ritual activities. In several cases fragments of pottery were associated with the structures: they indicate different and repeated visits to the area (from the Middle Pastoral to Early Garamantian ages).

A remarkable feature of this region is the presence of rock art evidence in direct association with the stone structures, from the slab showing two bovines at 00/556, to the all-round engraved pebble featuring a cow close to a stone platform (00/603), a rare masterpiece of portable art.

*Transect 1*

The transect is 8.5 by 2 km. No monuments were identified. Small quartzite outcrops are present, showing few traces of Middle Stone Age exploitation. The scarcity of archaeological findings in this area could be related, also during wetter periods, to its harsh environmental features: a strong upward wind due to the presence of the over 300m-high escarpment and the lack of water-related features were severe obstacles for the human presence.

*Transect 2*

The area (5x1 km) is located in the northern part of Wadi In Taghramt: both the bed and the low wadi banks were surveyed. In this area archaeological evidence are rare, limited to few historical and modern engravings (7 sites) and 6 small stone structures, probably related to Tuareg occupation.

*Transect 3*

This sector (9.5x2.5 km) encompasses a wide area limited to the west by Wadi In Erkni and to the east by Wadi In Taghramt. The wadis are narrow, deeply cut into the plateau, with minor meanders and several tributaries. The flat, black hamada is gently rolling showing few endorheic depressions. Some water reservoirs (gueltas) are also present. This physiographic unit was densely occupied during the Holocene. Archaeological contexts are mainly located in the vicinity of the wadi banks whereas the flat hamada was barely exploited.

The main archaeological evidence refers to the middle-late Holocene, from the tumuli of Late and Final Pastoral age to the more recent historical roadmarks and encampments. The few recorded rock art engravings confirm a recent frequentation of the area.

*Transect 4*

An area of 8.5x3 km located between Wadi Bedis and Wadi In Erahar was selected for the survey, as representative of a physiography made of narrow, deep, largely meandering wadis and flat hamada surfaces dotted by endorheic basins.

The transect encompasses the wide meander of Bedis and its surroundings, one of the densest areas identified. One hundred and thirty-six stone monuments (including 24 *corbeilles*), 12 occupation sites and more than 180 rock art panels were recorded. Almost all the rock art spots and most archaeological sites are located along the wadis’ banks, both in the Wadi Bedis and In Erahar meander. However, about 40% of the sites were recorded in the hamada between the wadis, often inside or close to small endorheic depressions or along small tributaries. The archaeological contexts cover a wide chronological framework, from the Pleistocene to historic and recent occupation, and a bulk of evidenced referable to the Pastoral Neolithic.

A detailed mapping covered an area of 1x0.3 km corresponding to the main Wadi Bedis meander, an area already defined as ‘*centre culturel*’ ([[8](#_ENREF_8)]: 110), where we selected the structures to excavate.

*Transect 5*

A smaller transect (2x1 km) was located in the eastern course of Wadi Bedis. Twenty archaeological monuments (5 *corbeilles*, 11 tumuli and other small recent stone structure) were recorded: 08/25 C1, a partially looted *corbeille*, appeared to be associated with a beautiful scene of cattle bearing water containers.

*Transect 6*

The southernmost surveyed area (2x1km) covers the confluence of three main wadis (Bedis, In Erahar, Takabart Kabort) into Wadi In Tullult. The area is representative of the terminal part of the fluvial systems. From a geomorphological point of view, Wadi In Tullult is large and its bed is formed by gravel bars and sand deposits. The surface of the hamada is deeper to the south and is much more undulated because it is dissected by a series of minor wadis; endorheic depressions are very rare. The archaeological evidences outlined also in this area a repeated human occupation from the Pastoral to recent times. The only context with *corbeilles* (07/55) was located on top of the plateau, in a sort of headland dominating the valley, above the famous rock art scene of ‘the new year procession’ (composed of several men with elaborated masks ([[9](#_ENREF_9)]: 36).

***References***

1. di Lernia S, Merighi F, Ricci F, Sivilli S (2002) From regions to sites: the excavation. In: di Lernia S, Manzi G, editors. Sand, Stones and Bones The Archaeology of Death in the Wadi Tanezzuft Valley (5000-2000 BP). Firenze: All'Insegna del Giglio. pp. 69-156.

2. Cremaschi M, di Lernia S (2000) Lasmo N-FC 174 Concession Area. The Messak Settafet Rescue Operation (Libyan Sahara): preliminary report. Department of Antiquities/ Cirsa: Tripoli and Rome.

3. di Lernia S (2006) Building monuments, creating identity: Cattle cult as a social response to rapid environmental changes in the Holocene Sahara. Quaternary International 151: 50-62.

4. di Lernia S, Cremaschi M (1997) Processing quartzite in central Sahara: a case-study from In Habeter IIIA-Wadi Mathendusc (Messak Settafet, Libya). Man and Flint: 225-232.

5. Corridi C (1998) Faunal remains from Holocene archaeological sites of the Tadrart Acacus and surroundings. Wadi Teshuinat Palaeoenvironment and Prehistory in South-western Fezzan (Libyan Sahara) 7: 89-94.

6. Ponti R (2003) Il tumulo di In-Habeter III (Sahara libico). Sahara 14: 161-166.

7. Lutz R, Lutz G (1992) From picture to hieroglyphic inscription. The trapping stone and its function in the Messak Sattafet (Fezzan, Libya). Sahara 5: 71-78.

8. Van Albada A, Van Albada AM (2000) La Montagne des Hommes-Chiens. Art Rupestre du Messak Libyen. Paris: Edition du Seuil.

9. Jacquet G (1988) Images d'un Sahara fertile. *Archéologia* 239: 34-41.
