# Supplementary material for: Inside the “African Cattle Complex”: Animal Burials in the Holocene Central Sahara
Source: PLoS One. 2013 Feb 20;8(2):e56879. doi: 10.1371/journal.pone.0056879 (PMC3577651; doi:10.1371/journal.pone.0056879)
Supplement: Table S4 — Main features of quartzarenite stone maces. (DOC) [file pone.0056879.s006.doc]

SI Table 4 – Main features of quartzarenite stone maces

| **Structure** | **layer** | **weight (g.)** | **length (mm)** | **as* width (mm)** | **h* width (mm)** | **description** | **wear traces** | **varnish** |
| --- | --- | --- | --- | --- | --- | --- | --- | --- |
| 07/28 C1 | surface | 3100 | 360 | 150 | 75 | stone mace with abrupt unidirectional retouch and undistinguished handle |  | both faces |
| 07/37 C1 | surface | 2000 | 300 | 178 | 72 | stone mace with invasive bidirectional retouch. Pointed active side and distinguished handle |  | both faces |
| 07/39 C3 | 3 | 1200 | 310 | 145 | 58 | stone mace with short bidirectional retouch. Rounded active side end distinguished handle |  | no |
| 07/39/C3 | 3 | 3100 | 355 | 210 | 72 | stone mace with short bidirectional retouch. Both rounded and pointed active side. Distinguished handle |  | no |
| 07/40 C1 | 3 | 4100 | 550 | 153 | 90 | short bidirectional retouched slab. Rectilinear active side. Two notches on the handle |  | no |
| 07/55 C1 | surface | 2200 | N.D. | 230 | N.D. | stone mace with invasive bidirectional retouch on the active side. Handle not preserved. |  | both faces |
| 07/110 C1 | 7 | 3400 | 340 | 184 | 85 | stone mace with invasive bidirectional retouch. Rounded active side and distinguished handle. |  | no |
| 07/110 C1 | 7 | 3300 | 325 | 170 | 45 | stone mace with invasive bidirectional retouch. Rounded active side and distinguished handle. Fractured |  | no |
| 07/110 C1 | surface | 700 | N.D. | N.D. | 60 | active side not preserved. Invasive bidirectional retouched handle. |  | both faces |
| 09/69 C1-C2 | surface | 3400 | 307 | 140 | 92 | stone mace with invasive bidirectional retouch. Rounded active side and distinguished handle. |  | both faces |
| 09/69 C1 | 6 | 3400 | 326 | 140 | 55 | stone mace with invasive bidirectional retouch. Rounded active side and undistinguished handle. |  | no |
| 00/556 | 5 | 3500 | 378 | 156 | 77 | stone mace in press with short bidirectional retouch. Rounded active side and undistinguished handle |  | no |
| 00/301 | 5 | 2300 | 264 | 170 | 55 | stone mace with invasive bidirectional retouch. Rounded active side and distinguished short handle | worn handle and wear related to thrusted percussion and resharpening of the active area. | no |
| 00/301 | 5 | 2400 | 300 | 170 | 55 | stone mace with invasive bidirectional retouch. Rounded active side and distinguished handle | worn handle and wear related to thrusted percussion and resharpening of the active area. | no |
| 00/328 | surface | 3500 | 340 | 165 | 40 | stone mace with short bidirectional retouch. Rounded active side and distinguished thin handle |  | both faces |
| 00/301 | 1 | 2600 | 215 | 180 | 70 | stone mace with invasive bidirectional retouch. Rounded active side. Handle not preserved. |  | both faces |

Key: as*, Active Side; h*, handle
